# Decolourization properties of pure phases in hydrated cement paste for anionic dyes in textile wastewater

**DOI:** 10.1016/j.heliyon.2025.e42231

**Published:** 2025-01-26

**Authors:** Martin Behringer, Harald Hilbig, Brigitte Helmreich, Alisa Machner

**Affiliations:** a Technical University of Munich, TUM School of Engineering and Design, Mineral Construction Materials, Franz-Langinger-Str. 10, 81245, Munich, Germany; b Technical University of Munich, TUM School of Engineering and Design, Urban Water Systems Engineering, Am Coulombwall 3, 85748, Garching, Germany

**Keywords:** Decolourization, Textile wastewater, Anionic dyes, Hydrated cementitious phases

## Abstract

The rise of fast fashion has increased the need for cost-effective adsorbents in textile wastewater treatment. Cementitious materials have shown promising decolourizing properties in anionic dye solutions. This study evaluates the decolourization properties of a selection of phases typically present in hydrated cement paste, such as calcium-(alumino)-silicate-hydrate (C-(A)-S-H) phases, hydrotalcite, and monocarboaluminate. Three anionic dyes were tested in combination with different salts, which are used in textile dyeing. Reactive Blue 19 was adsorbed more effectively than the other dyes. C-(A)-S-H phases performed best in basic environments, while layered structures excelled in acidic conditions, with monocarboaluminate reaching over 250 mg/g at pH 5. The presence of salts significantly affected adsorption, with MgSO_4_ mainly enhancing the decolourization up to 178 mg/g for monocarboaluminate and NaCl reducing capacities to a maximum adsorption of 95 mg/g for hydrotalcite. For hydrotalcite and monocarboaluminate, the effect of pH and presence of salts on the overall decolourization could be explained by a linear correlation between the zeta potential of the solid phases and their decolourization capacity. These findings provide valuable insights into the potential of cementitious materials as low-cost adsorbents in the treatment of coloured textile wastewater.

**Environmental implication**

This study highlights the environmental implications of using cementitious materials as low-cost adsorbents for harmful anionic dyes in textile wastewater. By demonstrating the effectiveness of phases such as C-(A)-S-H, hydrotalcite and monocarboaluminate under variable conditions, such as pH 5 and pH 11 as well as various dyeing salts, our research makes a significant contribution to environmental engineering. These materials offer sustainable solutions to mitigate the impact of hazardous dyes from the textile industry – a major contributor to water pollution – and provide a viable alternative to traditional wastewater treatment methods, reducing the environmental footprint of industrial dyeing processes.

## 1 Introduction

The rise of fast fashion has accelerated textile production, often at the expense of environmental sustainability [1]. An alarming consequence is that up to 15 wt% of the dyes used in textile manufacturing are discharged as effluent, causing significant water pollution [2,3]. The United Nations states that the textile dyeing industry is the second-largest water polluter globally, responsible for approximately 20 % of water pollution [4,5]. When not properly treated, these dyes pollute water sources and pose a hazard to aquatic life and human health [6]. Due to their complex chemical structures, high stability, and resistance to degradation, dyes are particularly challenging to remove, necessitating the development of effective treatment methods.

Numerous low-cost adsorbents, such as tree leaf powders [7] and banana and orange peels [[8], [9], [10]], among others [11,12], have been investigated for the removal of acid and reactive dyes, which are widely used in the textile industry [13]. The investigated adsorption processes primarily involve physical adsorption. Adsorption offers several advantages over other remediation approaches, such as chemical oxidation and biological treatments, as it is often more cost-effective to implement [14]. Additionally, adsorption and microbial methods can be combined to further enhance treatment capabilities [15]. However, adsorption also has limitations, including potential adsorbent saturation and the need for regeneration or disposal of used materials. Consequently, adsorption is one essential step within a multi-method approach required for effective wastewater treatment [16].

A previous study identified hydrated cement as a promising material for removing anionic azo dyes from wastewater [17]. Widely available and low-cost from concrete waste, hydrated cement can be utilized as granulates in textile dye outlets for pre-filtering wastewater. To allow optimization of this adsorption process, it is essential to investigate how the various phases within hydrated cement interact with dye molecules, thereby enabling the development of more effective and sustainable wastewater treatment solutions. Understanding these interactions also lays the groundwork for developing recycling systems to regenerate and reuse the dyed cementitious granulates, further enhancing the sustainability of this approach.

Hydrated cement phases are formed when cement reacts with water, resulting in the hardening of the mixture. The most prominent among these phases are calcium-silicate-hydrates (C-S-H), which can incorporate aluminium (C-A-S-H), as well as hydrotalcite or monocarboaluminate. While numerous studies have investigated the interactions between cementitious phases and various additives or contaminants [18], a significant knowledge gap remains regarding the specific adsorption mechanisms of anionic dyes on individual cementitious pure phases and their specific contribution to the overall dye uptake.

To address this gap, this study systematically evaluated the adsorption capacities of pure cementitious phases under various pH values and in different salt solutions, simulating real textile wastewater conditions. Specifically, we tested the dye adsorption capacity of calcium silicate hydrate (C-S-H) phases with calcium-to-silicon ratios (C/S) of 1.0 and 1.4, and calcium aluminosilicate hydrate (C-A-S-H) phases with aluminium-to-silicon ratios (A/S) of 0.05 and 0.1 (both with C/S = 1.0). In cement chemistry notation, C equals CaO, S = SiO_2_, A = Al_2_O_3_, and H = H_2_O. Additionally, we evaluated hydrotalcite, monocarboaluminate, Friedel's salt, brucite, and calcite on the three anionic dyes Reactive Blue 19, Acid Green 1, and Acid Orange 7 in various environments. We focused exclusively on anionic dyes, which are estimated to constitute up to 90 % of dyes present in wastewater [19]. By isolating these phases, the study aims to identify which phases contribute most significantly to dye adsorption and how environmental factors like pH and salt concentration influence this process. Advanced analytical techniques, including zeta potential measurements, qualitative X-ray powder diffraction, thermogravimetric analysis, and Fourier transform infrared spectroscopy, are employed to elucidate the underlying mechanisms governing dye adsorption.

These insights provide a more nuanced framework for predicting and optimizing dye removal in cement-based systems. While this research is primarily fundamental, focusing on basic interactions of the cementitious pure phases rather than immediate industrial application, it lays the groundwork for developing innovative, low-cost adsorbents made from cementitious materials that could lead to cleaner water bodies, improved ecosystems, and enhanced public health outcomes. By implementing these low-cost cementitious adsorbents in regions with significant textile dyeing industries, the innovation directly supports environmental sustainability by reducing pollutant levels in local water bodies and promoting healthier ecosystems, which aligns with the United Nations Sustainable Development Goals (SDGs) of Clean Water and Sanitation (SDG 6), Industry, Innovation, and Infrastructure (SDG 9), and Responsible Consumption and Production (SDG 12).

The main objectives of this work are to: (1) systematically evaluate the adsorption capacities of different cementitious phases in simulated textile wastewater, (2) determine the effect of environmental variables on these capacities, and (3) elucidate the underlying mechanisms in the interaction between the dyes and cementitious materials. This research is innovative in its approach to breaking down complex cementitious materials into their pure phases, providing a foundational understanding that can be applied to develop more effective, sustainable adsorbents for wastewater treatment.

## 2 Materials and methods

### 2.1 Materials

All materials used in this study were either synthesized in our laboratory or sourced from commercial suppliers. The materials synthesized include various cementitious phases such as calcium-silicate-hydrates (C-S-H), calcium-aluminate-silicate-hydrates (C-A-S-H), hydrotalcite, monocarboaluminate, and Friedel's salt. Detailed information regarding the materials used in the synthesis process can be found in the supplementary materials. Exceptionally, calcite and brucite were not synthesized but purchased. Other pure cementitious phases, such as portlandite and ettringite, were excluded because they completely dissolve at the concentrations investigated and are highly susceptible to carbonation, which alters their phases (e.g., portlandite converting to calcite).

#### 2.1.1 Synthesis of pure phases

Hydrotalcite (Mg_6_Al_2_CO_3_(OH)_16_·4H_2_O) was synthesized following the co-precipitation procedure initially proposed by Feitknecht [20,21]. A mixture of 42.0 g NaOH (Chemsolute, ≥99.0 %) and 30.0 g Na_2_CO_3_ (Chemsolute, ≥99.8 %) was added to 342 g of deionized water (<1 μS/cm) while maintaining intensive agitation. The resulting solution was transferred into a triple-necked 1-L flask. The temperature of the solution was raised to 35 °C using an oil bath. A second solution, a combination of 252 g deionized H_2_O, 92.3 g magnesium nitrate hexahydrate (Mg(NO_3_)_2_·6H_2_O) (Honeywell, 99 %), and 67.5 g aluminium nitrate nonahydrate (Al(NO_3_)_3_·9H_2_O) (Honeywell, ≥98.0 %), was prepared under rigorous agitation and dispensed into the flask at an average rate of 1.11 mL/min throughout the addition via a peristaltic pump. The solutions were homogenized at 340 rpm using an IKA RW 20 digital stirrer with a half-moon blade. After the preparation of the solution, the system was heated to 80 °C, to accelerate nucleation and growth of the crystals. The target temperature was reached after approximately 90 min due to system inertia and maintained for 15 h, to ensure complete crystallization and optimal formation of the layered double hydroxide phases. The resultant slurry was poured into 50-mL conical tubes and centrifuged at 11,000 rpm for 5 min. The solids were repeatedly washed and centrifuged until the Na^+^ content in the supernatant was below 600 mg/L, as determined by inductively coupled plasma optical emission spectroscopy (ICP-OES). The samples were then dried in a vacuum oven at ∼200 hPa and 120 °C for approximately 20 h and stored in a desiccator until further testing.

C-S-H phases featuring two distinct C/S-ratios, 1.0 (CSH1.0) and 1.4 (CSH1.4), were synthesized by combining calcium oxide (CaO), silica fume (Aerosil 200) (Evonik, ≥99.8 %), and distilled, deionized water in varying proportions. CaO was derived from the calcination of calcium carbonate (CaCO_3_) (Chemsolute, ≥99.5 %) over 15 h at 1000 °C. For CSH1.0, 9.65 g CaO, 10.4 g SiO_2_, and 900 mL freshly distilled water were filled into a 1000 mL HDPE wide mouth bottle in an argon atmosphere, to prevent the carbonation of CaO. The bottles were sealed and constantly agitated in the overhead mixer at 7 rpm for 21 days. A similar procedure was followed for the C/S ratio 1.4, using 11.3 g CaO and 8.67 g SiO_2_. The suspensions were then filtered, and the remaining solids were transferred to Petri dishes and dried for eight weeks in an N_2_ atmosphere over silica gel, which was replaced five times a week.

The initial step in producing C-A-S-H phases began with synthesizing calcium aluminate (CA). This involved mixing 12.7 g CaCO_3_ and 12.9 g aluminium oxide (Al_2_O_3_) (Roth, ≥99.0 %) and heating the mix in Pt/Au crucibles at temperatures up to 1400 °C for 5 h. After cooling in a desiccator, the product was ground for 40 s in a tungsten-carbide horizontal disk mill. This was followed by three additional grinding sessions of 3 min each in a mortar agate mill with maximum pestle pressure. Concurrently, CaO was produced by calcining CaCO_3_, while CO_2_-free water was prepared by distillation.

For the synthesis of C-A-S-H phases, the target C/S ratio was 1.0, with target A/S ratios of 0.05 (CASH0.05) and 0.10 (CASH0.1). To prepare CASH0.05, 9.21 g of CaO was mixed with 10.1 g of SiO_2_ and 0.67 g of CA. For the preparation of CASH0.1, 8.79 g of CaO was mixed with 9.91 g of SiO_2_ and 1.30 g of CA. The compounds were mixed with deionized, distilled water at a w/s ratio of 45. The compounds were placed into 1000 mL wide-mouth bottles (HDPE) in an argon atmosphere, sealed, and mixed for 21 days using an overhead mixer at 7 rpm. Then, the resulting solid was filtered off and stored in a desiccator under a slight vacuum for eight weeks, with silica gel replaced five times per week.

To produce Friedel's salt (3CaO·Al_2_O_3_·CaCl_2_·10H_2_O), tricalcium aluminate (C_3_A) was initially synthesized. This was achieved by blending 58.5 g of CaCO_3_ (Chemsolute, ≥99.5 %) and 19.9 g of Al_2_O_3_ (Roth, 99.0 %) in an agate mortar grinder. The blended materials were heated to 1400 °C in a Pt/Au crucible for 8 h. The resulting solid was initially ground in a disk mill with a tungsten carbide vessel for 20 s and subsequently in an agate mortar grinder for three rounds, each lasting 3 min, with maximum pestle pressure. The material was sintered twice to eliminate unwanted phases such as lime and mayenite (cf. Fig. S6 in the electronic supplementary material). The phase assemblage was verified using X-ray diffraction (XRD). Friedel's salt was then synthesized by combining the prepared C_3_A with calcium chloride dihydrate (CaCl_2_·2H_2_O) (Chemsolute, ≥99.5) in a 1:1 M ratio, precisely 48.6 g C_3_A and 26.4 g CaCl_2_·2H_2_O. To achieve a water-to-solid ratio of 10, two 1000 mL wide-mouth bottles were each filled with 750 mL of freshly distilled, room-temperature water and 75 g of the solid components. The mixing process was carried out in an argon-filled glovebox, and the bottles were then fixed in an overhead mixer, operating at 7 rpm for 30 days. The solids were filtered off and dried in an N_2_-flushed desiccator over silica gel.

For monocarboaluminate (3CaO·Al_2_O_3_·CaCO_3_·nH_2_O), calcium aluminate (CA) was prepared by blending 63.33 g of CaCO_3_ (Chemsolute, ≥99.5 %) with 64.52 g of Al_2_O_3_ (Roth, ≥99.0 %). The mixed reagents were homogenized in the overhead mixer at 7 rpm for 1 h before calcination at 1000 °C overnight. After calcination, the powder was subjected to three grinding cycles in a mortar mill, each lasting 3 min. The milled powder was then heated to 1400 °C for 5 h. Post-sintering, the solid material was cooled in a desiccator over silica gel with potassium hydroxide (KOH), serving as a CO_2_ trap. The cooled solid was again ground in the mortar mill for three cycles of 3 min each. QXRD analysis was carried out on a sample to determine the CaO content. After repeating the sintering step, the material was cooled in a desiccator over silica gel with a KOH-CO_2_ trap for approximately 1 h. The solid materials were combined in an argon-filled glovebox: 29.9 g of CA was mixed with 14.0 g of calcium hydroxide (Ca(OH)_2_) (Chemsolute, ≥96.0 %) and 18.9 g of CaCO_3_ in a 1-L PET bottle. Finally, 700 mL of distilled water was added to the mixture in an argon atmosphere, sealed, and subjected to overhead mixing (7 rpm) for 14 days. After mixing, the suspension was filtered, and the solids were dried in a desiccator over silica gel under a light vacuum.

All synthesized solids were dried until they reached a constant mass. They were then ground three times for 3 min in a mortar mill with maximum pestle pressure, manually sieved (<63 μm – woven mesh), and stored in a desiccator at a slight vacuum.

#### 2.1.2 Dyes

To represent a wide variety of anionic dyes in the study, we used 3 different dyes, which belong to different dye classes with various molecular sizes. Therefore, for the decolourization experiments, Remazol Brilliant Blue R (Sigma Aldrich; chemical formula: C_22_H_16_N_2_Na_2_O_11_S_3_; molar mass: 626.54 g/mol; water solubility: 1 mg/mL), also known as Reactive Blue 19 (RB19), was used as a reactive dye and belongs to the anthraquinone dye class. Naphthol Green B (Alfa Aesar; chemical formula: C_30_H_15_FeN_3_Na_3_O_15_S_3_; molar mass: 878.47 g/mol; water solubility at 20 °C: 160 mg/mL), also known as Acid Green 1 (AG1), was used as an acid dye and belongs to the nitro dye class. Acid Orange 7 (AO7) (abcr; chemical formula: C_16_H_11_N_2_NaO_4_S; molar mass: 350.32 g/mol; water solubility at 30 °C: 116 g/L) was used as an acid dye and belongs to the mono-azo dye class.

### 2.2 Methods

#### 2.2.1 Decolourization experiments with UV–VIS spectroscopy

Before the decolourization experiments, we evaluated the impact of different amounts of solid additions (0.1, 0.2, 0.5, and 1 g of each pure phase) to a 100 mg/L RB19 dye solution to determine the optimal amount for effective decolourization. A 100 mg/L dye solution was prepared using 500 mL of deionized water. The initial pH values were adjusted to 5 or 11 using HCl and NaOH and monitored during the decolourization experiments with a Schott ProLab 400 pH meter featuring an SI Analytics pH-combination electrode N65. These pH values represent typical pH ranges observed in textile wastewaters [[22], [23], [24]]. To represent common additives in the dyeing process, various salts (MgSO_4_, Na_2_SO_4_, and NaCl) were added at a concentration of 0.5 mol/L to the dye solution [4,25,26]. Although these model solutions do not fully mimic actual textile wastewater, they can hint at how the presence of salts changes the decolourization behaviour.

In each experimental setup, 200 mg of the synthesized pure phases were added to the 500 mL of dye solution. The progression of decolourization was tracked with a specially equipped PerkinElmer Lambda 465 UV–VIS spectrometer, using an 8-channel flow-through cell with an automated sample changer unit and an ALS PCP 151L Metering Pump. The wavelength was measured in the range of 300 nm–800 nm. For the analysis, specific wavelengths were selected for different dyes: 585 nm for Reactive Blue 19, 600 nm for Acid Green 1, and 322 nm for Acid Orange 7. An initial absorbance measurement was performed on the dye(-salt) solution at a predetermined concentration of 100 mg/L before the addition of the solid phases. Subsequently, the solids were added to the solution, and periodic absorbance measurements were conducted. The first ten measurements were conducted minutely, followed by ten measurements every 5 min and 12 measurements every 10 min for 3 h and 32 measurement points. The decolourization was calculated based on the initial absorbance (c_0_) ratio to the absorbance at each time point (c) as determined from the UV/VIS spectra. To prevent obstruction in the hoses and analysis chamber, 10 μm UHMW polyethylene cannula filters (Erweka) were used.

#### 2.2.2 Zeta potential

The zeta potential measurements complemented the decolourization experiments using a Dispersion Technology 1200 instrument, which employs the electroacoustic method. Zeta potential is determined through the colloidal vibration current (CVI) methodology.

For accurate CVI assessment, it is essential to eliminate the ion vibration current (IVI) from the total vibration current (TVI). This requires quantifying the ion vibration background before each measurement, which is then subtracted from the TVI. Such a process is particularly critical in analyzing large particles, specifically those exceeding 5 μm in diameter (d_50_). The inherent limitations of this instrument prevent it from storing measured backgrounds, requiring them to be measured before each data collection session. This dependency introduces a higher margin of error in the absolute values obtained. Consequently, our primary focus shifts to the ability of the zeta potential to reveal trends and variations when comparing different environmental conditions.

To reduce the impact of the ion vibration current (IVI), the solid concentration was increased to 5 wt%, in contrast to the decolourization experiments. Before each measurement, the ion vibration background was measured. This background was prepared by replicating the experimental setup but removing the solid beforehand by filtration, measuring the effect of the dissolved components in the liquid. During the measurement, the pH of the samples was monitored using a Metrohm LL-Solitrode pH electrode. A constant temperature of 20 °C was maintained throughout the experiment. The experiments were carried out in a 50 mL glass beaker with a magnetic stirrer. 1.0 g of solid was diluted in 20 mL of deionized water to prepare the dye solution. The experiments included three different pH levels: highly alkaline (pH 13), equilibrium pH, and acidic (pH 5), with HCl and NaOH employed for pH adjustment of the liquid phase.

#### 2.2.3 X-ray diffraction

Powder X-ray diffraction (XRD) analysis was employed to determine possible structural differences in the solids before and after decolourization. A Bruker D8 Advance X-ray diffractometer equipped with a Cu-tube (1.5418 Å wavelength) and a LYNXEYE_XE_T detector were used for the analyses. The XRD measurements covered a 2-Theta range of 5–70° with a step size of 0.02° and a measurement time of 0.2 s/step. The measurements were conducted on a Si low-background sample holder due to the small sample size. The data obtained were evaluated using the DIFFRAC software.EVA V4.3.1 by Bruker AXS.

#### 2.2.4 ^29^Si and ^27^Al nuclear magnetic resonance spectroscopy

All Silicon (^29^Si) nuclear magnetic resonance spectroscopy (NMR) measurements were conducted with a Bruker Avance 300 spectrometer (magnetic field strength 7.045 T; resonance frequency 59.63 MHz) in single pulse magic angle spinning (SP MAS) mode. The ground pure phases were packed into 7 mm zirconia rotors and spun at 5 kHz for ^29^Si-NMR before the decolourization experiments. About 2000 scans were recorded with a repetition time of 45 s. The ^29^Si chemical shifts were referenced relative to tetramethylsilane (TMS). The ^27^Al NMR spectroscopy was carried out with a Bruker AvanceNeo 500 spectrometer (Bruker, Germany) with a magnetic field strength of 11.747 T (resonance frequency for ^27^Al: 130.308 MHz). The measurements were performed using the SP MAS technique. The samples were placed in a 4 mm zirconia rotor and spun at 12 kHz with a repetition time of 0.5 s, recording 2000 scans. The ^27^Al chemical shifts were referenced relative to [Al(H_2_O)_6_]^3+^.

The deconvolution was performed using the software OriginPro 2019, following the procedure described by Krüger et al. [27] fitting to a pseudo-Voigt shape limiting the FWHM to 3 and a fixed profile shape of 0.5. The average chain lengths were calculated following Richardson [28].

#### 2.2.5 Brunauer-Emmett-Teller analysis

The specific surface area was measured using the BELSORP-mini II by MicrotracBEL with N_2_ as an adsorbate gas. Before the measurement, the ground pure phases were dried in a high vacuum (10 Pa) at 40 °C for 24 h using the Belprep vac II to ensure moisture removal. Measurements were conducted at 77 K using a Dewar's vessel filled with liquid nitrogen. The specific surface area was calculated using the Brunauer-Emmett-Teller (BET) method [29].

#### 2.2.6 Analysis of the solutions

Ion concentrations in the solutions were determined using ICP-OES (PerkinElmer Avio 500). Solubilities were calculated as the sum of the main ions in the filtered aqueous phase.

pH values were measured with a pH-combination electrode from SI Analytics, Type A 162 2M-DIN-ID. The equilibrium pH of all pure phases was correlated with solutions of 1 wt% in deionized water.

## 3 Results and discussion

### 3.1 Characterization of pure phases prior to decolourization experiments

#### 3.1.1 Reference to figures

All characterization data of the synthesized phases (cf. Figs. S1–S9 and Table S1) are presented in the supplementary material, and decolourization data is available in the repository at https://mediatum.ub.tum.de/1748399 (https://doi.org/10.14459/2024mp1748399). The commercial calcite and brucite were confirmed to possess sufficient purity through qualitative XRD, Fourier-transform infrared spectroscopy (FT-IR) and ICP-OES analyses.

#### 3.1.2 C-(A)-S-H phases

The synthesized C-(A)-S-H phases were characterized by ICP-OES, ^29^Si-NMR and XRD. ICP-OES data were used to calculate the molar C/S ratio and, for C-A-S-H phases, the molar A/S ratio (cf. Table 1). The targeted C/S ratios were slightly lower than the measured ratios. The measured A/S ratios match the targeted A/S ratios.

**Table 1:** Targeted and measured molar ratios of C-(A)-S-H phases as measured by ICP-OES.

ID	Targeted C/S ratio	Targeted A/S ratio	Measured C/S ratio	Measured A/S ratio
CSH1.0	1.0	–	1.12	–
CSH1.4	1.4	–	1.50	–
CASH0.05	1.0	0.05	1.05	0.05
CASH0.1	1.0	0.10	1.03	0.09

In ^29^Si-NMR, both C-S-H phases showed two major peaks at ∼79 ppm (Q^1^) and ∼85 ppm (Q^2^_(p)_) (cf. Fig. S1), showing a slight chemical peak shift to less negative values with an increased C/S ratio. CSH1.0 showed a higher intensity peak at the Q^2^ position compared to CSH1.4 and vice versa for the Q^1^ sites at ∼79 ppm Q^1^ sites are located on the end of chains or as isolated pairs. Therefore, a rising ratio of Q^1^ to Q^2^ indicates a shorter mean chain length, which has also been reported in various studies before [[30], [31], [32]]. The mean chain length (2∗(Q^1^ + Q^2^)/2) for the investigated phases is 7.26 for CSH1.0 and 2.77 for CSH1.4, which matches average values reported in the literature [[30], [31], [32]] and confirms the trend that a higher C/S ratio indicates a shorter mean chain length.

The C-A-S-H phases also show two major peaks at ∼79 and ∼85 ppm (cf. Fig. S2). Additionally, the shoulder at ∼82.5 ppm is attributed to Q^2^_b_ or Q^2^(1Al) [28,33]. The calculated mean chain length depends strongly on the peak positions. However, with the described Q^2^(1Al) peak at 82.5 ppm following Richardson [28], the mean chain lengths (MCL) were calculated to be 14.17 and 23.23 for CASH0.1 and CASH0.05 respectively. These MCLs are higher than those reported in the literature [33,34]. This may be attributed to slight carbonation of the phases, as shown by TGA (cf. Fig. S8), leading to decalcification. XRD analysis showed no portlandite, which likely forms at higher C/S ratios [30].

^27^Al shows a broad peak between 60 and 70 ppm. Next to the main peak, the peak at ∼10 ppm can be attributed to CAH-phases, meaning that not all aluminium is incorporated into the structure. This is most likely because, after only 21 d of synthesis, the solution has not yet reached equilibrium [33].

#### 3.1.3 Hydrotalcite

Hydrotalcite was characterized using XRD, ^27^Al-NMR, ICP-OES, FT-IR and BET (cf. supplementary material). All peaks observed in the XRD spectrum are attributable to hydrotalcite. The basal spacing – and consequently the peak position – is influenced by the anions in the interlayer structure. The main central peak position at 11.575° 2θ indicates interstratification in the interlayer occupied by OH^−^ and CO_3_^2−^ [35]. A possible interstratification is further supported by the higher order (00l) reflections that do not strictly follow the c_0_/2, c_0_/3 pattern [36]. However, the peaks at higher 2θ angles – especially those ranging from 37 to 58 – are notably broad. This broadening is associated with many stacking faults in the sheet-like structure [37]. No crystalline Mg(OH)_2_ or Al(OH)_3_ was detected. ^27^Al-NMR data showed a symmetric peak at 8.98 ppm with spinning sidebands at ∼102 ppm. Elemental analysis via ICP-OES showed an approximate Mg/Al ratio of 4. FT-IR spectroscopy revealed a peak between 3,600 and 2,600 cm^−1^ with an additional broad feature at 3500 cm^−1^, indicating O-H stretching. A sharp peak at 1360 cm^−1^ with a small shoulder at 1410 cm^−1^ corresponds to the asymmetric stretching vibration of CO_3_^2−^, split into two peaks [38]. The measured peak is shifted slightly to a lower wavenumber than that reported by Zhang et al. [39], suggesting a symmetry reduction [38]. Between 1000 and 600 cm^−1^, several overlapping peaks correspond to Mg/Al - O absorption bands. BET surface area was measured at 75.3 m^2^/g.

#### 3.1.4 Monocarboaluminate

Monocarboaluminate was characterized by XRD, ^27^Al-NMR, ICP-OES, FT-IR and BET (cf. supplementary material). Qualitative XRD identified the primary peak (010) at a d-spacing of 7.58 (11.660 2θ) with a secondary peak (020) at a d-spacing of 3.789. Traces of gibbsite and slight carbonation were observed, as evidenced by a distinct calcite peak at 29.364 2θ. ^27^Al-NMR spectroscopy revealed a single, symmetric peak at 8.45 ppm, with spinning sidebands at ∼102 ppm. FT-IR spectroscopy detected several minor peaks between 3700 and 3300 cm^−1^, attributable to O-H stretching. For CO_3_^2−^ stretching, two peaks were observed: one peak at 1412 cm^−1^ and a second at 1360 cm^−1^, along with a broad shoulder ranging from 1550 to 1450 cm^−1^. Additional small peaks were observed from 1050 to 600 cm^−1^. The Ca/Al ratio was determined via ICP-OES as 2.5. BET surface area was determined as 11.7 m^2^/g.

#### 3.1.5 Friedel's salt

Friedel's salt was characterized by XRD, ^27^Al-NMR, ICP-OES, FT-IR, and BET (cf. supplementary material). Qualitative XRD analysis identified the primary peak at 11.21° 2θ. A minor calcite peak was also observed, indicating slight carbonation. No other crystalline phases were detected. ^27^Al-NMR revealed a single, symmetric peak at 8.30 ppm.

### 3.2 Decolourization experiments

After the synthesis and characterization of the pure phases, the decolourization experiments were conducted. Decolourization data can be evaluated using surface area (mg/m^2^) and mass (mg/g). Given the standard practice in decolourization studies, a mass-based measurement was chosen. Nonetheless, Table 2 shows the measured surface areas (BET), which can be used to calculate the adsorption capacity in mg/m^2^. In addition to the surface area, the solubility of the pure phases can affect their decolourization properties. The calculated solubility values via ICP-OES for all tested cementitious phases, as given in Table 2, apply to pure water conditions. We did not emphasize phase solubility in our decolourization experiments, considering the entire system collectively, thereby making it difficult to differentiate between diluted and undiluted constituents. The equilibrium pH values (cf. Table 2) provide additional insights into the chemical environment of each phase.

**Table 2:** BET specific surface area and calculated solubility. The solubility was calculated as the sum of the main elements in the filtered aqueous phase quantified by ICP-OES, excluding the loss on ignition. The equilibrium pH of all pure phases (1 wt%) in deionized water was measured with a pH-combination electrode by SI Analytics Type A 162 2M-DIN-ID.

Phase	BET surface area [m^2^/g]	Standard deviation (n = 3–4)	Calculated solubility in water [mg/L]	Equilibrium pH
CSH1.0	21.1	5.7	42.1	–
CSH1.4	32.0	6.1	46.9	11.4
CASH0.05	31.6	0.4	19.9	10.8
CASH0.1	31.9	2.3	22.5	–
Brucite	15.2	1.5	8.73	10.8
Calcite	0.9	0.3	7.77	9.6
Hydrotalcite	75.3	9.4	3.76	9.0
Monocarboaluminate	11.7	0.2	81.7	11.3
Friedel's salt	8.3	1.3	171	11.7

Table 3 gives an overview of the percentage of dye removal per each 0.2 g solid in a 500 mL dye solution (100 mg/L) after 3 h in different environments. This dye concentration was chosen as it resulted in a measurable change and aimed to simulate real-world scenarios, such as highly coloured textile industry effluent diluted into rivers or other wastewater.

**Table 3:** Percentage of decolourization of 0.2 g tested pure phases in various conditions within a 500 mL dye solution (100 mg/L) after 3 h; n = 3–6.; complete decolourization equals 100; concentration of salts was 0.5 mol/L.

Dye Material/Environment	AG1	AO7	RB19					
	pure	pure	pure	pH 5	pH 11	MgSO_4_	NaCl	Na_2_SO_4_
Brucite	<1	<1	14	8	9	32	<1	22
Calcite	<1	<1	13	5	6	14	9	12
CASH0.05	<1	<1	13	4	13	39	9	8
CASH0.1	<1	1	13	6	13	Not measured		
CSH1.0	<1	<1	14	5	13			
CSH1.4	<1	1	13	5	7	15	11	5
Friedel's salt	<1	<1	<1	66	4	66	9	10
Hydrotalcite	10	24	31	43	11	20	38	25
Monocarboaluminate	11	7	8	>100	14	71	4	58

**Fig. 1:**
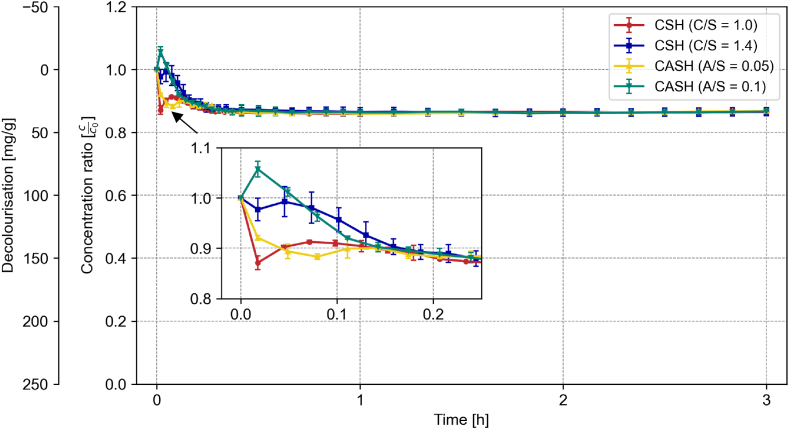
Corrected concentration ratio of each 0.2 g C-(A)-S-H phases in 500 mL at 100 mg/L of RB19 over 3 h during the decolourization experiment; error bars = standard deviation [n = 3].

Initial absorbance measurements, from t = 0 min (immediately before the addition of the solid material) to t = 5 min, were unstable, displaying a transient absorption peak in the UV/VIS spectrum. This peak diminished after several minutes. To account for this problem, control measurements were conducted in the absence of the dye. The measured absorption curves were subsequently subtracted from the corresponding sample absorption curve to correct for any interference. The correction procedure was applied to all measurements. Occasionally, discrepancies in peak height were observed between the control and experimental measurements. This even happened with the corrected values, resulting in concentration ratios exceeding 1.0 or leading to inflated decolourization capacities in the initial minutes (cf. Fig. 1).

#### 3.2.1 Interaction of cementitious phases in pure RB19 solution

Considering the numerous factors affecting the decolourization potential, we initially investigated a simplified system. To begin, the cementitious pure phases were examined as adsorbents for Reactive Blue 19 (RB19) in pure water. Fig. 1 shows the decolourization of the synthesized C-(A)-S-H phases over a period of 3 h.

All tested C-(A)-S-H phases reached similar decolourization values around 0.86 c/c_0_, corresponding to an approximate adsorption capacity of 34.5 mg/g (±1.5) of RB19 per gram of solid material. The fluctuations in the measurement within the first 15 min are affected by the initial transient absorption peak, as described above. A plateau is reached after approximately 20 min. Due to the similar decolourization behaviour, all further tests were conducted only with one C-S-H (C/S = 1.4) and C-A-S-H (A/S = 0.05, C/S = 1.0) phase.

**Fig. 2:**
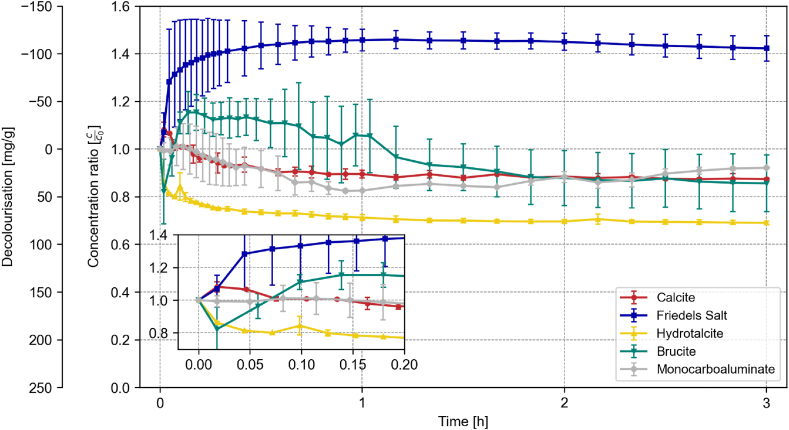
Decolourization of each 0.2 g of calcite, Friedel's salt, hydrotalcite, brucite, and monocarboaluminate over 3 h in a 500 mL solution with a RB19 concentration of 100 mg/L. Error bars = standard deviation [n = 3–6].

The correction factor was only partially effective for brucite and Friedel's salt compared to the C-(A)-S-H phases, resulting in pronounced fluctuations in the absorbance data – especially within the first hour (cf. Fig. 2). However, given the transient nature of the fluctuations, the final adsorption capacities for all phases are comparable, with the exception of Friedel's salt.

Under these conditions, brucite showed an adsorption capacity of 36.1 mg/g, while calcite demonstrated a slightly lower capacity of 31.6 mg/g. Nonetheless, considering the surface area, calcite provides significantly less surface area relative to the other phases (cf. Table 2). Consequently, the measured dye uptake of calcite in mg/m^2^ is significantly higher than for the other phases.

Friedel's salt shows an increase in absorbance after the addition to the dye solution in Fig. 2, suggesting a corresponding increase in dye concentration within the solution. However, no additional dye was added, and measurement errors were statistically eliminated (n = 6). The measured absorbance spectrum, recorded at each time point, reveals an upward shift in the spectrum without any visible additional peaks. This was not the case in the correction measurement, suggesting that the dye and the solid react in a way that is active in UV/VIS. ICP-OES analysis of the liquid phase after 20 and 40 min showed a decline of Cl, Ca, and Al compared to the correction measurement without dye. This suggests that the dye and Friedel's salt form complexes affecting the absorption measurement. Consequently, an accurate quantitative measurement of the adsorption capacity is not feasible.

Monocarboaluminate exhibits a slow decolourization during the first hour, with the absorbance ratio c_o_/c oscillating around 0.9, which calculates to 19.8 mg/g adsorbed dye. This behaviour may imply that the dye adsorption is unstable on the solid surface.

Hydrotalcite decolourizes the solution effectively within the first 15 min and then slowly stabilizes at a c_0_/c value of 0.68, corresponding to a significantly higher adsorption capacity of 79.5 mg/g for RB19 compared to the monocarboaluminate.

In addition to RB19, the interactions of two other dyes, AG1 and AO7, were also investigated using pure cementitious phases, as detailed in Table 3 and Figs. S10 and S11 of the electronic supplementary material. For AG1, the decolourization capacities were generally lower than those for RB19. Only monocarboaluminate showed an increased decolourization capacity, rising from 19.8 mg/g with RB19 to 27.8 mg/g with AG1. Hydrotalcite showed a similar capacity at 26.0 mg/g, significantly lower than the 77.5 mg/g observed with RB19. CASH0.05 demonstrated a capacity of less than 1 mg/g compared to the 33.2 mg/g recorded for RB19.

In contrast, for AO7, CASH0.05 exhibited no measurable decolourization capacity, whereas both monocarboaluminate and hydrotalcite were effective. Notably, hydrotalcite demonstrated a substantially higher capacity for AO7 at 59.0 mg/g compared to 17.6 mg/g for monocarboaluminate, while both phases exhibited similar decolourization capacities when using the other acid dye, AG1. XRD and FT-IR analysis confirmed that all phases maintained structural integrity post-decolourization, suggesting that the specific properties of each dye influence differences in decolourization efficacy.

#### 3.2.2 Impact of the pH value

The conditions in actual textile wastewater differ substantially from those in pure systems. Several studies investigated the dependence of the adsorption capacity on the pH value of the solution [7,9,11,12]. Thus, all materials were tested at low (pH 5) and high (pH 11) pH values as well as the equilibrium pH (cf. Table 2). These values were chosen because they represent limits for commonly reported values in textile wastewater [22]. Also, most cementitious phases are not stable at very low pH values [40]. Hydrotalcite's lower equilibrium pH, compared to monocarboaluminate and Friedel's salt, may account for its enhanced decolourization efficacy. However, the relatively low pH was not a decisive factor for calcite due to its inherently limited decolourization capacity. We hypothesize that while pH value is a key factor in decolourization potential, it is not the only one, as evidenced by the limited decolourization efficacy of calcite.

At pH 11, CASH0.05 demonstrated an adsorption capacity of 33.3 mg/g, similar to its performance in the pure system (33.2 mg/g), with only minimal NaOH required for pH adjustment. At pH 5, the adsorption capacity substantially decreases to 20.5 mg/g. For CSH1.4, the adsorption capacity at pH 11 value was lower (18.0 mg/g) compared to the value at equilibrium pH (33.5 mg/g), as HCl was needed to adjust for the equilibrium pH value of 11.4. At pH 5, its adsorption capacity reduced further to 13.2 mg/g. This trend suggests that lower pH values negatively impact the adsorption efficacy of C-(A)-S-H phases. A possible explanation is that at low pH, these phases tend to have higher coordination, leading to larger molecules of silica gel [40] and, therefore, less surface area for dye adsorption.

A correlation between a low pH and a reduced adsorption capacity was also seen for calcite and brucite. In the pure system, their adsorption capacities decreased from 31.6 to 36.1 mg/g at equilibrium pH, respectively, to 12.5 and 18.7 mg/g at pH 5. Notably, the data for brucite exhibited substantial variability during the measurements, diminishing its reliability. Nevertheless, there was a clear trend toward lower adsorption capacity.

**Fig. 3:**
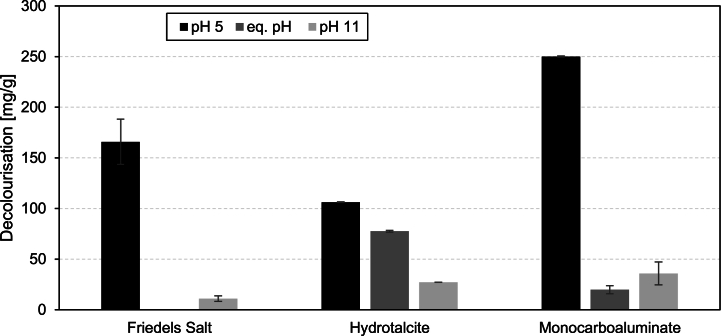
Decolourization capacity of Friedel's salt, hydrotalcite, and monocarboaluminate after 3 h in a 500 mL solution with a RB19 concentration of 100 mg/L; n = 3–6; error bars = standard deviation.

Conversely, lower pH values enhanced the decolourization efficacy of hydrate phases such as Friedel's salt, hydrotalcite, and monocarboaluminate, as distinctly shown in Fig. 3. Differences for C-(A)-S-H, calcite and brucite are less pronounced and therefore not shown (cf. Table 3 and linked data repository).

A strong correlation between pH value and decolourization capacity can be seen for hydrotalcite, where the decolourization capacity increases with lower pH values and decreases with higher pH values. In the case of Friedel's salt at pH 5 and pH 11, significant initial fluctuations were observed, which stabilized after a few minutes. At pH 11, Friedel's salt showed negligible decolourization, with an adsorption capacity of 10.9 mg/g after 3 h. No value is depicted for Friedel's salt's decolourization capacity in equilibrium pH, as no decolourization was measured. Conversely, at pH 5, its adsorption capacity surged to 166 mg/g for Reactive Blue 19 after 3 h. Even more remarkable was the decolourization performance of monocarboaluminate. At pH 11, an adsorption capacity of 35.87 mg/g was recorded after 3 h. Remarkably, at pH5, the solution was completely decolourized within 16 min, resulting in an adsorption capacity exceeding 250 mg/g. This capacity exceeds that of materials such as powdered activated carbon in a similar study, where the predicted maximum uptake of RB19 was found to be 106 mg/g after 8 h [41]. All three of these phases share a layered structure consisting of positively charged main layers and negatively charged interlayers [42,43]. Previous studies pointed out that these phases can intercalate ions within their interlayer regions [35], potentially accounting for the high adsorption capacities observed. However, no indication of intercalation was found, neither before nor after the decolourization, as the main peak position did not change, as shown by XRD analysis (cf. Fig. S14). In contrast, the post-decolourization analysis for monocarboaluminate revealed that no monocarboaluminate was detectable in the solid phase. Due to the low pH, monocarboaluminate was dissolved entirely, and gibbsite and small amounts of calcite were exclusively visible in the XRD (cf. Fig. S13).

Given that textile wastewaters are typically alkaline and that the decolourization efficacy of some cementitious pure phases decreases at higher pH, using cementitious materials for decolourization could present challenges in real-world applications. However, our study shows that certain phases, such as C-(A)-S-H, maintain their adsorption capacity under alkaline conditions. This indicates that the selection of suitable raw materials for hydrated cements or concrete may serve to mitigate the challenges associated with elevated pH values in textile wastewater.

#### 3.2.3 Impact of salts

Building on our pH impact analysis, we further investigated how commonly used dyeing salts, such as MgSO_4_, Na_2_SO_4_ and NaCl, affect the decolourization capacity of various phases. For C-S-H phases, the addition of MgSO_4_ and NaCl had only marginal impacts on the adsorption capacity compared to the results obtained in the pure system. The adsorption capacity of CSH1.4 increased slightly from 33.5 mg/g in the pure system to 36.8 mg/g with the addition of MgSO_4_ and decreased to 27.0 mg/g with NaCl. Conversely, Na_2_SO_4_ reduced the decolourization efficacy, resulting in an adsorption capacity of 11.9 mg/g after 3 h. In the pure system, CASH0.05 had an adsorption capacity of 35.3 mg/g, which decreased to 22.7 mg/g with NaCl and 20.4 mg/g with Na_2_SO_4_. Remarkably, the presence of MgSO_4_ almost tripled the adsorption capacity for CASH0.05, resulting in an adsorption capacity of 98.9 mg/g. The observed adsorption capacities for calcite were 34.4, 22.5, and 30.9 mg/g in the presence of MgSO_4_, NaCl, and Na_2_SO_4_, respectively. These values showed slight variation compared to the reference system without salts (31.6 mg/g).

**Fig. 4:**
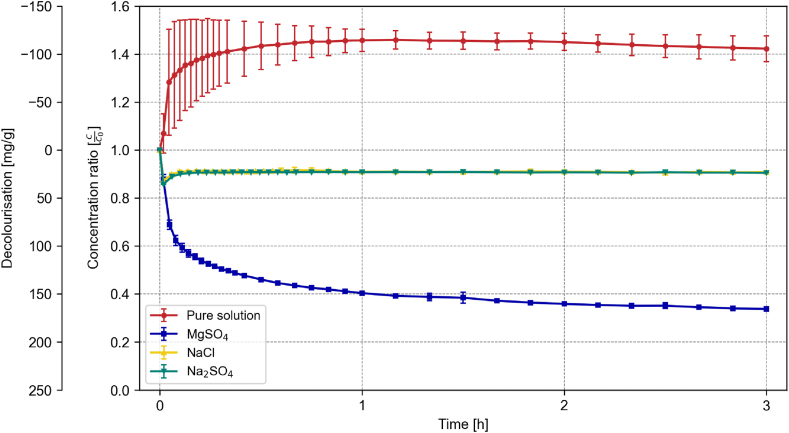
Development of the decolourization and concentration ratio of 0.2 g Friedel's salt in 500 mL of a 100 mg/L RB19 solution over the first 3 h in the presence of 0.5 M MgSO_4_, NaCl, or Na_2_SO_4_ compared to the pure solution; n = 3; error bars = standard deviation.

For Friedel's salt, the impact of salt presence on the decolourization capacity is more significant compared to the other phases studied. As illustrated in Fig. 4, the decolourization profiles differed remarkably across the three salt solutions. Interestingly, the upward shift in the adsorption spectrum observed upon solid addition is absent in salt-containing solutions.

**Fig. 5:**
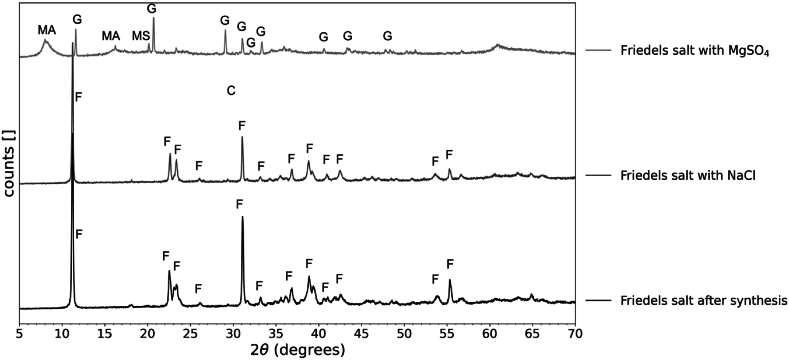
XRD Analysis of Friedel's salt before decolourization (bottom) compared to after decolourization in NaCl (middle) and after decolourization in a MgSO_4_ environment (top). F = Friedel's Salt; C = Calcite; MA = Magnesium Aluminum Sulfate Hydroxide Hydrate; MS = Magnesium Sulfate Hydrate; G = Gypsum.

MgSO_4_ considerably enhances the adsorption capacity of Friedel's salt, reaching 166 mg/g, in stark contrast to the 23.2 and 23.9 mg/g observed for NaCl and Na_2_SO_4_, respectively. Post-decolourization analysis of the solid using XRD revealed that Friedel's salt remains stable in NaCl solutions. In contrast, exposure to MgSO_4_ leads to the complete dissolution of Friedel's salt, forming gypsum, magnesium aluminium sulfate hydroxide hydrate, and magnesium sulfate hydrate, as shown in Fig. 5.

**Fig. 6:**
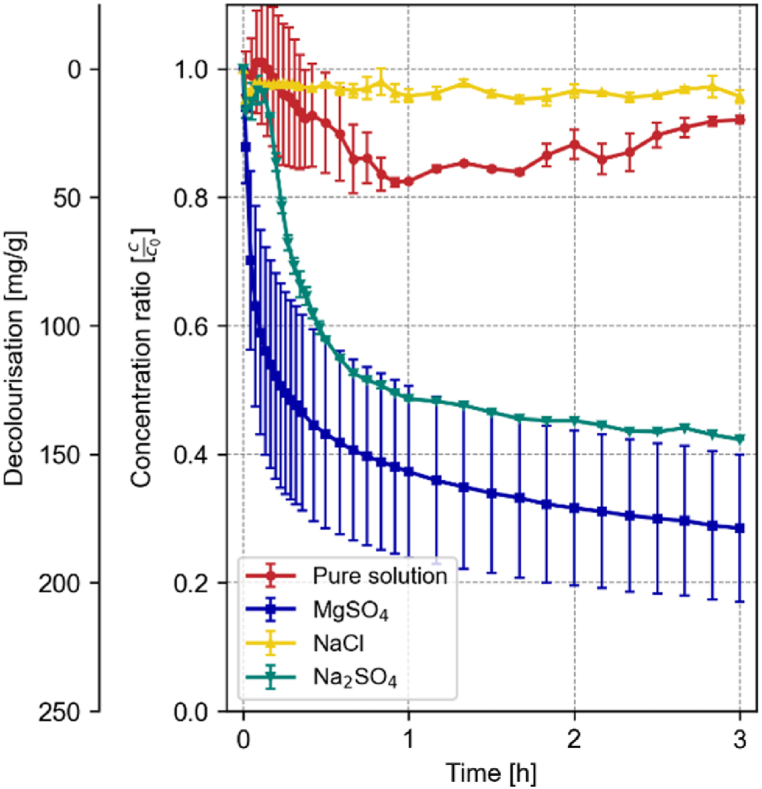
Development of the decolourization and concentration ratio of 0.2 g Monocarboaluminate in 500 mL of a 100 mg/L RB19 solution over the first 3 h in the presence of 0.5 M MgSO4, NaCl, or Na2SO4 compared to the pure solution; n = 3; error bars = standard deviation.

Similarly, for monocarboaluminate (19.8 mg/g in the pure solution), the presence of salts greatly affects the adsorption capacity. While NaCl slightly decreases the adsorption capacity to 10.8 mg/g, sulfate-containing salts greatly enhance the dye uptake to 144 and 179 mg/g for Na_2_SO_4_ and MgSO_4,_ respectively (cf. Fig. 6).

**Fig. 7:**
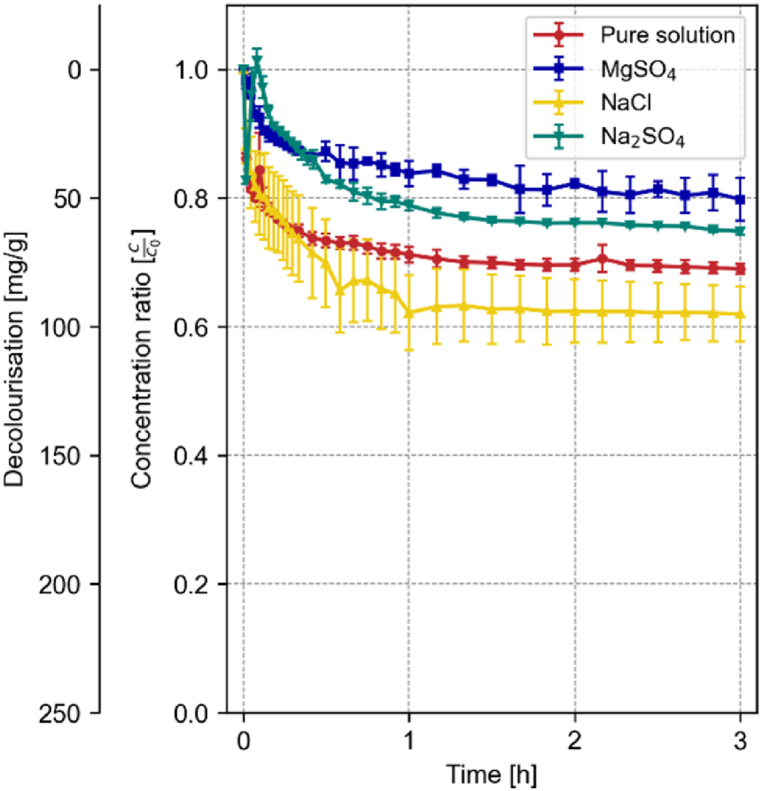
Development of the decolourization and concentration ratio of 0.2 g hydrotalcite in 500 mL of a 100 mg/L RB19 solution over the first 3 h in the presence of 0.5 M MgSO_4_, NaCl, or Na_2_SO_4_ compared to the pure solution; n = 3; error bars = standard deviation.

However, the capacity of hydrotalcite (77.6 mg/g in the pure solution) is lowest when MgSO_4_ is dissolved in the solution with 50.6 mg/g, followed by Na_2_SO_4_ (62.9 mg/g). In contrast, the presence of NaCl enhances the uptake to 95.02 mg/g (cf. Fig. 7).

The enhanced dye adsorption capacity observed in the presence of MgSO_4_ can be attributed to chemical interactions between MgSO_4_ and the cementitious pure phases. A high concentration of MgSO_4_ in the solution causes the decomposition of the phases and the formation of gypsum and other magnesium-containing phases [44]. In these cases, the Mg^2+^ ions replace the Ca^2+^ ions in the hydrated phases.

In contrast, the presence of NaCl or Na_2_SO_4_ did not enhance the decolourization capacity of the phases, except for monocarboaluminate and brucite. Na^+^ is less likely to replace the Ca^2+^ sites in the hydrated phases [45], therefore no calcium is free to react with the Cl^−^ or the SO_4_^2−^ to form new adsorption sites. As well, cementitious phases are less likely to form gypsum in the presence of Na_2_SO_4_, than in solutions containing MgSO_4_ [44].

The presence of these ions significantly affected the adsorption capacity of all phases, as evidenced by the changes in decolourization capacities (cf. Fig. 4, Fig. 6, Fig. 7). This suggests that interfering ions can either enhance or inhibit adsorption, depending on the specific interactions between the ions, dye molecules, and adsorbent surfaces.

Different adsorbents, as studied by Balci et al. (2022), demonstrated decolourization capacities ranging from 30 mg/g to 265 mg/g for Reactive Blue 19 (RB19) under comparable conditions of dye concentration, adsorbent dosage, and equilibrium time [41]. In our study, the adsorption capacities of cementitious pure phases fall within this range, with monocarboaluminate on the upper end achieving adsorption capacities exceeding 250 mg/g at pH 5 and C-(A)-S-H phases on the lower end with an adsorption capacity of around 32 mg/g without salts or pH adjustments.

However, a direct comparison may be misleading because some of the tested cementitious phases undergo decomposition during the decolourization process. This decomposition can alter the structural integrity and surface properties of the adsorbents, potentially enhancing or diminishing their adsorption capacities. Therefore, while our cementitious phases perform comparable to other low-cost adsorbents, the underlying mechanisms contributing to their adsorption efficiencies may differ due to these phase transformations.

### 3.3 Zeta potential

Dye adsorption typically occurs on the surface of a solid and is dependent on a complex interplay of electrostatic and van der Waals forces [46]. Thus, zeta potential measurements are crucial for understanding the interactions between surfaces that determine the adsorption of dyes. They provide critical insights into the effect of surface charge on adsorption efficiency, particularly between positively charged surfaces and negatively charged dyes.

All three dyes, Reactive Blue 19, Acid Green 1, and Acid Orange 7, exhibit a net negative charge in aqueous solutions due to the dissociation of positively charged sodium ions from the parent dye molecule [47]. The negatively charged dye molecule tends to adsorb on surfaces possessing positive charges due to electrostatic interactions. Due to van der Waals forces, however, it is also possible for a negatively charged dye to adhere to negatively charged surfaces. Zeta potential measurements provide significant information concerning these fundamental mechanisms by evaluating the surface charge, or more specifically, the electrostatic potential at the shear plane of the material. The shear plane is the boundary layer within the electrical double layer that surrounds particles in solution. This electrostatic potential present in the boundary layer often indicates the measured surface charge of the particle. Nonetheless, comparing absolute zeta potential values between different studies is problematic. This is because zeta potential is affected by factors such as solvent chemistry, ionic strength, ionic species, and measurement techniques, such as electroacoustic and electrophoretic methods [46,48]. As such, zeta potential measurements are primarily of interest for intra-study comparisons.

**Fig. 8:**
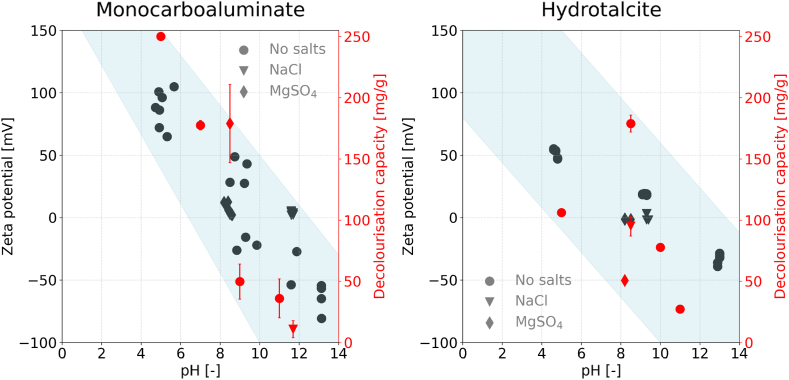
Measured zeta potential of hydrotalcite (right) and monocarboaluminate (left) in different conditions compared to the decolourization capacity. The black markers represent the zeta potential, while the red markers display the decolourization capacity of the respective measurement. The circle-shaped markers show the results where no salts were added over a pH range; the triangle-shaped markers show all samples measured in a NaCl solution, and the diamond-shaped markers were measured in a MgSO_4_ environment. The blue highlighted area illustrates the correlation between zeta potential and decolourization capacity as a guide to the eye. Error bars = standard deviation.

A high zeta potential indicates a strong positive charge on the particle's surface, thereby enhancing the likelihood of adsorption of the negatively charged RB19 molecule. This effect is especially pronounced for monocarboaluminate, which shows a significant decolourization capacity at low pH. Fig. 8 displays the zeta potential measurements for monocarboaluminate and hydrotalcite at pH 5, the equilibrium pH, and pH 13, compared with the decolourization capacity observed after 3 h on the second y-axis.

At lower pH levels, the zeta potential shows high values at approximately +80 mV (±20), which linearly decreases to negative values around −70 mV at a pH of 13. This trend aligns with the decolourization capacity measurements, demonstrating a minimum fivefold increase of decolourization at a pH 5 compared to that observed at pH 11. The measured zeta potential values are on the limit of what has been previously reported for various highly charged particles [49,50]. The background subtraction as an intermediate step introduces an additional source of potential error, which may result in slight deviations from the absolute values. The high decolourization capacity when monocarboaluminate is added to an anionic dye solution at pH 5, however, cannot be attributed to the phase monocarboaluminate, considering its degradation under such conditions as shown by XRD (cf. Fig. S12).

The measured zeta potential measurements, where 0.5 mol/L of either MgSO_4_ or NaCl were added to the deionized water, reveal low zeta potential values for both salts. The measured values in the presence of MgSO_4_ are slightly higher, with a mean of 6 mV, compared to the values for NaCl, with approximately 4 mV for NaCl. When comparing the decolourization capacity, a considerably higher decolourization capacity can be seen when MgSO_4_ is present compared to NaCl. The slight difference in zeta potential is unlikely to explain the high difference in decolourization capacity. However, the precise cause remains unknown to the authors and requires additional detailed study.

Similar results were observed for hydrotalcite (Fig. 8), where a high positive zeta potential at low pH transitions to a negative zeta potential at higher pH levels. The decolourization capacity, similar to that of monocarboaluminate, increases at low pH levels and decreases at higher pH levels. Hydrotalcite, however, exhibits a roughly linear zeta potential under conditions from pH 5 to pH 11. At the same time, the crystalline parts of the phase are destroyed at low pH, as measured by XRD (cf. Fig. S13). In the presence of salts, the absolute zeta potential values are notably low. While the presence of MgSO_4_ results in an approximate zeta potential of −1 mV, the presence of NaCl results in marginally higher values of +1.5 mV. This pattern is mirrored in the decolourization data, with hydrotalcite showing increased dye adsorption in the presence of NaCl. Still, the magnitude of the difference makes it unlikely that the zeta potential is the only cause for differences in the decolourization capacity. Similar to the measurements for monocarboaluminate and hydrotalcite, the authors have not determined the precise cause for this.

Both, hydrotalcite as well as monocarboaluminate, possess a layered structure, which is characterized by its positively charged layers balance by the interlayer anions and water molecules [44]. This arrangement results in a higher surface charge, compared to other cementitious pure phases, contributing to a higher zeta potential and therefore a higher decolourization capacity for anionic dyes.

**Fig. 9:**
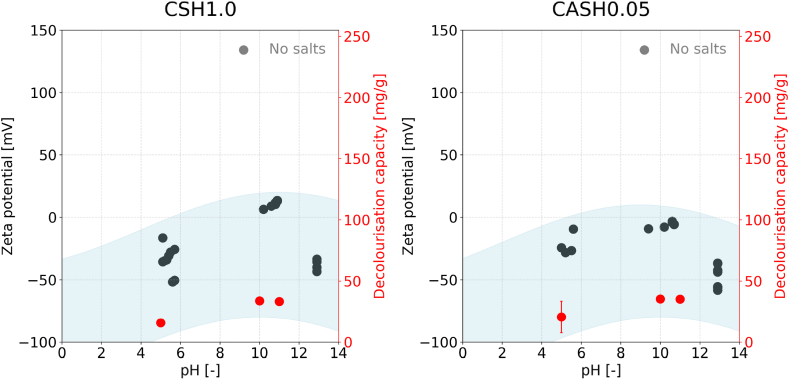
Measured zeta potential for CSH1.0 (left) and CASH0.05 (right) over the range of pH 5 to pH 13 compared to the respective decolourization capacity. The black markers represent the Zeta potential, and the red markers display the decolourization capacity of the respective measurements. The blue highlighted area illustrates the connection between zeta potential and decolourization capacity as a guide for the eye. Error bars = standard deviation.

Conversely, no significant variation in decolourization capacity was observed with pH changes in the C-(A)-S-H phases (Fig. 9). The zeta potential measurements for these phases do not seem to follow a linear trend but, instead, a parabolic pattern, where the highest decolourization capacity is observed at the equilibrium pH, with lower capacities at both higher and lower pH levels compared to the equilibrium pH value. This might be misleading as the C-(A)-S-H phases are unstable over the tested pH range. When reaching pH 8 and lower, the phases transform into an amorphous silica gel [33,40]. The zeta potential is negative at low pH values, slightly increasing to a minimal absolute value at equilibrium pH, then decreasing again to a more negative value upon adding NaOH. Due to the phase change, no consecutive fitting line will match, and more measurement points at various pH values are needed.

The decolourization effect of concrete cannot be solely attributed to a specific phase; rather, it results from the complex interplay among all existing phases and is significantly influenced by their chemical environment. A logical next step would be to conduct similar analyses on cementitious systems, which would consist of various phases. Understanding the decolourization capacities of individual pure phases can help to predict or calculate the overall decolourization capacity of cementitious materials based on their composition.

### 3.4 Practical applications

This study serves as a foundational step toward the development of cementitious granulates as a globally availably low-cost adsorbent for the textile dyeing industry. While the pure cementitious phases investigated are not intended for direct application and are merely a first step in the design stage to real wastewater treatment scenarios, the insights gained enhance our understanding of the interactions between hydrated cement phases and anionic dyes. These results guide the development and refinement of cementitious materials, setting the stage for subsequent studies aimed at incorporating cementitious granulates into industrial wastewater treatment systems and assessing their performance in real-world scenarios, like the decolourization of batik wastewater, to improve environmental sustainability in localities with a major textile dyeing industry [51]. Implementing such materials could provide sustainable and economically viable solutions for reducing pollutant levels in the short term while supporting concrete integration into a circular economy. This approach offers a cost-effective alternative to activated carbon, which is often expensive to produce and use.

From an economic standpoint, cementitious materials derived from waste concrete or industrial by-products present a cost-effective alternative to traditional adsorbents such as activated carbon, which incurs high production and activation expenses [52]. However, hydrated cement and concrete, particularly when combined with blast furnace slag, can leach harmful metals into the environment upon contact with water. Future studies should evaluate whether the advantages of using these materials outweigh their potential environmental risks. Compared to other low-cost adsorbents like agricultural waste, estimated at approximately $0.90 per kilogram [52], cementitious materials can be manufactured at even lower costs [53]. Utilizing demolition waste can reduce production costs even further, making these materials more affordable than many other low cost adsorbents.

## 4 Conclusion

This study advances the understanding of cementitious materials as low-cost adsorbents for the removal of anionic dyes from wastewater, addressing critical environmental challenges associated with the textile industry. By systematically evaluating pure cementitious phases – including C-(A)-S-H with varying C/S ratios, hydrotalcite, monocarboaluminate, Friedel's salt, calcite, and brucite – we have elucidated their distinct decolourization properties under diverse conditions, breaking down complex cementitious materials into their individual phases and identifying specific adsorption mechanisms that were previously unclear. As a result, our work lays the groundwork for the future development of optimised cementitious granules to improve their effectiveness and sustainability as adsorbents in the textile dyeing industry.

The decolourization capacity of the tested pure cementitious phases varies significantly, from less than 1 mg/g (several phases; cf. Table 3) to over 250 mg/g for monocarboaluminate at pH 5.

Lower pH levels (pH 5) enhance the decolourization in most cementitious hydration phases, especially monocarboaluminate, while having little or negative effect on C-(A)-S-H, calcite, and brucite. The instability of most phases at low pH values means that an unknown part of their decolourization capacity is due to their decomposition products.

The presence of various ions from dyeing salts is critical to adsorption performance. MgSO₄ generally boosts decolourization across phases except hydrotalcite by enabling the replacement of Ca^2^⁺ with Mg^2^⁺, resulting in the formation of more effective calcium sulfate structures that create additional adsorption sites. On the other hand, Na₂SO₄ and NaCl reduce adsorption efficacy as Na⁺ ions do not substitute Ca^2^⁺ efficiently and instead compete for adsorption sites, thereby diminishing dye uptake. A notable correlation exists between zeta potential and decolourization; a high positive zeta potential correlates with a tendency of higher decolourization capacity for the tested anionic dyes in the various tested environments. However, the absolute values of zeta potential are not directly comparable across different phases.

While this study focuses on the fundamental adsorption properties of pure cementitious phases, we acknowledge that the reusability and regeneration of adsorbents are crucial factors for practical wastewater treatment applications. Future research will explore the reusability of cementitious materials, such as cementitious granulates, to assess their performance over multiple adsorption-desorption cycles.

By focusing on fundamental interactions between pure cementitious phases and anionic dyes, this research lays the groundwork for future studies aimed at optimizing cementitious materials for practical applications. Future research should explore the scalability of using cementitious granulates in real-world wastewater treatment facilities and investigate the reusability and regeneration of these adsorbents to ensure their long-term viability and economic feasibility. Additionally, integrating adsorption with other treatment methods could further enhance the efficiency and sustainability of wastewater management systems.

## CRediT authorship contribution statement

**Martin Behringer:** Writing – original draft, Visualization, Validation, Methodology, Investigation, Formal analysis, Data curation, Conceptualization. **Harald Hilbig:** Writing – review & editing, Validation, Supervision, Methodology, Funding acquisition, Conceptualization. **Brigitte Helmreich:** Writing – review & editing, Validation, Supervision, Methodology, Funding acquisition, Conceptualization. **Alisa Machner:** Writing – review & editing, Validation, Supervision, Methodology, Conceptualization.

## Funding

We want to thank the 10.13039/501100001659German Research Foundation (DFG) for their financial support (project number: 495389990).

## Declaration of competing interest

The authors declare that they have no known competing financial interests or personal relationships that could have appeared to influence the work reported in this paper.
