# Supplementary material for: Decolourization properties of pure phases in hydrated cement paste for anionic dyes in textile wastewater
Source: Heliyon. 2025 Jan 26;11(4):e42231. doi: 10.1016/j.heliyon.2025.e42231 (PMC11869016; doi:10.1016/j.heliyon.2025.e42231)
Supplement: Multimedia component 1 [file mmc1.docx]

Supplementary material to:

Decolourisation properties of pure hydrated cementitious phases for anionic dyes in textile wastewater

Martin Behringer^1*^, Harald Hilbig^1^, Brigitte Helmreich^2^, Alisa Machner^1^

^1^Technical University of Munich, TUM School of Engineering and Design, Professorship for Mineral Construction Materials, Franz-Langinger-Str. 10, 81245 Munich, Germany

^2^Technical University of Munich, TUM School of Engineering and Design, Chair of Urban Water Systems Engineering, Am Coulombwall 3, 85748 Garching, Germany

*Corresponding author: Martin Behringer, [martin.behringer@tum.de](mailto:martin.behringer@tum.de)

# Synthesis protocols:

## CASH-phases:

**Synthesis begin:** 07.02.2024

**Equipment:**

- 2 Pt/AU 95/5 FKS Crucible No. 44VX 150 ml

- High-temperature laboratory furnace - rhf (Carbolite 1500 °C)

- Calcium carbonate (Chemsolute 2414, min. 99.5%)

- Silicon dioxide (Aerosil 200, fumed silica)

- Aluminum oxide (Roth 9420.3, min. 99%, calcined, pure)

- Argon-filled glovebox

- Overhead mixer (Heidolph REAX 20)

- 6 wide mouth bottles (LABSOLUTE® WIDE MOUTH BOTTLES; Material: HDPE; Capacity: 1000 ml)

- 598 Rundfilter (filter paper circles, d = 125 mm, Schleicher & Schuell)

- Mortar grinder (Retsch RM 200, agate)

**Procedure:**

CASH phases were produced with a C/S ratio of 1.0 and A/S ratios of 0.05 (CASH0.05), 0.1 (CASH0.1), and 0.2 (CASH0.2) of which only CASH0.05 and CASH0.1 were used for the decolourisation experiments. In advance, CA was produced. Therefore, two Pt/Au crucibles were filled with 12.667 g CaCO_3_ and 12.903 g Al_2_O_3_ each. Before adding, both solids were homogenised in an agate mortar. The crucibles were then heated to 1000 °C at a rate of 4 K/min and calcined for about 12 hours. After the heating period, the temperature was increased to 1400 °C at a rate of 20 K/min and maintained for 5 hours. The solid was then cooled down and first ground in a horizontal disk mill (tungsten/carbide) for 40 seconds and subsequently for 3 minutes three times in the mortar mill. The heating process was then repeated, and the solid was cooled down and ground again using the same method. After each grinding step, a qualitative XRD was performed to ensure no (or only small amounts of) Al_2_O_3_ or Mayenite was left.

CaO was produced by heating CaCO_3_ to 1000 °C at 10 K/min for 15 hours. Meanwhile, CO_2_-free water was made by distilling pure water (<1 µS/m) and storing it upside down in 1 L PET bottles. The water was filled into 1 L PET wide mouth bottles (900.0 ml per bottle), sealed with parafilm, and stored upside down for approximately 20 hours to prevent CO_2_ from entering. Subsequently, 9.91 g SiO_2_ (MIX 1 - A/S = 0.05) and 9.51 g SiO_2_ (MIX 2 - A/S = 0.1) were weighed into 1 L PET wide-mouth bottles.

The CaO sample was removed from the furnace and cooled in a desiccator with KOH over silica gel. After approximately 30 minutes, the sample was cooled to room temperature and placed into the argon-filled glovebox, where the CO_2_-free water and SiO_2_ had been placed. In the glovebox, the CaO was manually ground in an agate mortar in small doses for approximately 5 minutes each. The CA was also ground in the glovebox for about 5 minutes. 9.21 g CaO, 10.1 g SiO_2_, and 0.666 g CA were mixed for a total solid amount of 20 g in 900 ml water (w/b = 45) for CASH0.05. 8.79 g CaO, 9.91 g SiO_2_, and 1.30 g CA were mixed for CASH0.1, and 7.99 g CaO, 9.51 g SiO_2_, and 2.50 g CA were mixed for CASH0.2 in 900 ml freshly distilled and deionized water.

After dismounting, the solid was filtered using 598 filters (Schleicher & Schuell; d = 125 mm) and then stored in Petri dishes in a N_2_-filled desiccator over silica gel and KOH as a second CO_2_ trap. The silica gel was changed five times a week. After each replacement, the desiccator was flushed with N_2_ for 2 minutes, and the drying period lasted for eight weeks. The material was then ground in a mortar mill three times for 3 minutes each and sieved through a 63 µm sieve. The solid was then stored in snap-cap vials and sealed with parafilm.

## CSH-phases:

**Synthesis Begin:** 09.03.2023

**Equipment:**

- 2 Pt/AU 95/5 FKS Crucible No. 44VX 150 ml

- High-temperature laboratory furnace - rhf (Carbolite 1500 °C)

- Calcium carbonate (Chemsolute 2414, min. 99.5 %)

- Silicon dioxide (Aerosil 200, fumed silica)

- Argon-filled glovebox

- Overhead mixer (Heidolph REAX 20)

- 6 wide mouth bottles (LABSOLUTE® WIDE MOUTH BOTTLES; Material: HDPE; Capacity: 1000ml)

- 598 Rundfilter (filter paper circles, d = 125 mm, Schleicher&Schuell)

**Procedure:**

Two times, approximately 60 g CaCO_3_ was filled into the Pt/Au crucibles and heated in the furnace to 1000 °C at a rate of 10 K/min for about 15 hours. Meanwhile, CO_2_-free water was produced. Therefore, pure water (<1 µS) was distilled and stored upside down in 2 L PET bottles. The water was filled into 1 L PET wide-mouth bottles (900 ml per bottle), sealed with parafilm, and stored upside down for approximately 20 hours to prevent CO_2_ from entering. Subsequently, three times 10.4 g SiO_2_ and three times 8.67 g SiO_2_ were weighed in 1 l PET wide-mouth bottles. The CaO sample was removed from the furnace and cooled in a desiccator with KOH over silica gel. After approximately 30 min, the sample was cooled to room temperature and placed into the argon filed glovebox, where the CO_2_-free water and SiO_2_ had been placed. In the glovebox, the CaO was manually ground in an agate mortar in small doses for approximately 5 min each. Then, three times, 9.65 g CaO was weighed in and placed into the three bottles containing 10.4 g SiO_2_ (Mixture 1). Three times 11.3 g CaO were added to the second three bottles containing 8.67 g SiO_2_ (Mixture 2). The water was added to the powder, and the PET bottles were sealed with tape. Mixture 1 results in a C/S ratio of about 0.9, and Mixture 2 results in a C/S ratio of 1.4. (The C/S ratio of 1.0 was produced in a second round.) The bottles were then mounted in the overhead mixer at 7 rpm for 21 days (end date: 31.03.2023). After dismounting, the solid was filtered using 598 Filter (Schleicher&Schuell; d = 125 mm) and then stored in Petri dishes, which were placed into a N_2_-filled desiccator over silica-gel and KOH as a second CO_2_ trap. The silica gel was changed five times a week. After every replacement, the desiccator was flushed with N_2_ for 2 min.

After five weeks of drying, the Petri dishes were weighed daily when exchanging the silica gel to ensure constant mass. After reaching mass constancy, the solid was ground in a mortar mill for 3 min 3 times and then sieved through a 63 µm sieve.

## Hydrotalcite:

**Synthesis Begin:** 21.03.2023 12:00

**Equipment:**

- Peristaltic pump (LabCraft HYDRIS 05, serial number: 2165050902)

- 3-neck flask (1 liter)

- Laboratory stirrer (IKA RW 20 digital)

- half-moon stirring blade (BOLA GT-Glas-Rührwellen, PTFE/Glas)

- 600 ml Beaker

- heating plate (ICA RCT basic)

- lab scissor jack

- rod thermometer (IKA ETS D5)

- Oil bath (silicon oil)

**Procedure:**

First, 42.0 g of NaOH and 30.0 g of Na_2_CO_3_ were prepared in separate petri dishes. A 600 ml beaker was filled with 342 g of deionised H_2_O (<1 µS). Na_2_CO_3_ was slowly added to the beaker under vigorous stirring, followed by the gradual addition of NaOH under vigorous stirring. The resulting solution was then poured into a flask.

An oil bath was heated until the liquid in the flask reached a temperature of 35 °C, with the heating plate set to 65 °C. This heating process took approximately 1 hour. 252 g of deionised H2O (<1 µS) was added to another 600 ml beaker for the second solution. Subsequently, 92.3 g of magnesium nitrate hexahydrate and 67.5 g of aluminum nitrate nonahydrate were added to the beaker while stirring. The initial weights of these compounds were set at 20 wt.% above the stoichiometric amount to ensure equal pumping until the end, with the stoichiometric amount controlled by the speed of the peristaltic pump.

After the initial 1-hour waiting period for Solution 1 to heat up, Solution 2 was added at a rate of 1.11 ml/min, with the pump set to 18.9 and stirring set to 340 rpm. This addition resulted in the formation of small white flakes in the solution, which eventually turned into a white viscous mass. After 4 hours, the pump was shut down, and the hose was removed. The heating plate was then set to 200 °C, and the rod thermometer was adjusted to a target temperature of 80 °C. Due to the system's inertia, the heating plate produced an error approximately every 3 minutes, as the thermometer failed to recognise a change in temperature and shut down the heating plate for safety reasons. Each time this error occurred, the heating plate was turned back on, and the target temperature was reset. After about 30 minutes, the oil bath began heating at a sufficient speed, preventing further errors. The target temperature of 80 °C was reached after approximately 90 minutes.

The heating period continued until the following day (~15 h), when the heat was turned off, and the hydrotalcite liquid was poured from the flask into a beaker. The hydrotalcite solution was then divided into twelve 50 ml conical tubes (with a total mass of each tube of 62 g without lid) and centrifuged for five minutes at 11,000 rpm. The liquid was separated and analysed using ICP-OES to check the Na^+^ concentration. Pure water was added to the solids in the tubes (total mass 58 g without lid). The mixture was stirred with a glass rod for about 2 minutes per tube to separate the resulting Na+ from the hydrotalcite. The samples were centrifuged again, and the process was repeated three times, resulting in four centrifugation cycles.

After centrifugation, the solid was placed in a petri dish and heated at 120 °C in a vacuum oven at ~200 hPa for about 20 hours. The dried solid was then ground in a mortar mill three times for 3 minutes each and sieved through a 63 µm mesh. The resulting powder was stored in snap-cap glass vials, sealed with parafilm, and stored in a desiccator at a low vacuum over silica gel.

## Monocarboaluminate:

**Synthesis Begin:** 13.01.2023

**Equipment:**

- 2 Crucibles Pt/AU 95/5 FKS Cup No. 44VX 150 ml

- Aluminum oxide (Roth 9420.3, min. 99 %, calcinated)

- Calcium carbonate (Chemsolute 2414, min. 99,5 %)

- Mortar grinder (Retsch RM 200, agate)

- High-temperature laboratory furnace - rhf (Carbolite 1500 °C)

- XRD diffractometer (Bruker D8 Advance)

- Argon-filled glovebox

- Overhead mixer (Heidolph REAX 20)

- 6 wide mouth bottles (LABSOLUTE® WIDE MOUTH BOTTLES; Material: HDPE; Capacity: 1000ml)

- Ca(OH)_2_ (Roth, min 96 %, p.a.)

**Procedure**:

CA was produced by mixing 63.3 g CaCO_3_ with 64.5 g Al_2_O_3_ twice in a Pt/Au crucible. The reagents were homogenised in the overhead mixer for one hour. The homogenised powder was calcined overnight at 1000 °C with a 4 K/min heating rate. After calcination the powder was ground three times for 3 min in the mortar mill. The ground powder was then heated to 1400 °C for five hours at a 20 K/min heating rate. After heating, the solid was cooled down in a desiccator with KOH as CO_2_ Trap over Silicagel. The material was then again ground three times for 3 min in the mortar mill. A sample was analysed with XRD to obtain the CaO content. Then, the burning process was repeated. The material was then cooled down in a desiccator with KOH as a CO_2_ trap over silica gel for approximately 1 hour.

The solids were then mixed in an Ar-filled glovebox. Therefore, 29.9 g CA was mixed with 14.0 g Ca(OH)2 and 18.9 g CaCO_3_ in a 1 l PET bottle. At last, 700 ml of distilled water was added to the mix and then put into the overhead mixer for 14 d.

After mixing, the solid was filtered and dried in a desiccator over silica gel at a low vacuum of 0.07- 0.08 MPa. When dried, the solid was ground three times for 3 min in a mortar mill and subsequently sieved through a 63 µm sieve and stored in snap cap vials for further analysis.

## Friedel’s salt:

**Synthesis Begin:** 20.03.2023

**Equipment:**

- 2 Pt/AU 95/5 FKS Cup No. 44VX 150 ml

- Aluminum oxide (Roth 9420.3, min. 99 %, calcinated)

- Calcium carbonate (Chemsolute 2414, min. 99,5 %)

- Vibratory disk mill with tungsten carbide vessel (Herzog Maschinenfabrik, Type HSM 50)

- Mortar grinder (Retsch RM 200, agate)

- High-temperature laboratory furnace - rhf (Carbolite 1500 °C)

- XRD diffractometer (Bruker D8 Advance)

- Calcium chloride dihydrate (Chemsolute 2461.0500, min 99.5%)

- Tricalcium aluminate (C3A - synthesised)

- Argon-filled glovebox

- Overhead mixer (Heidolph REAX 20)

- Six wide mouth bottles (LABSOLUTE® WIDE MOUTH BOTTLES; Material: HDPE; Capacity: 1000ml)

**Procedure:**

The synthesis began with the production of C_3_A. Two batches of 58.4 g CaCO_3_ and 19.9 g Al_2_O_3_ were homogenised in an agate mortar and then filled into a Pt/Au crucible. The crucibles were placed in a furnace at room temperature, and the temperature was increased from 10 K/min to 1000 °C, with a dwell time of 8 hours. After this period, the furnace temperature was raised to 1400 °C at a rate of 20 K/min, with another dwell time of 8 hours. After heating, the samples were removed from the furnace and cooled.

The cooled material was ground in a vibratory disk mill with a tungsten carbide vessel for 20 seconds. The resulting powder was ground in a mortar grinder three times for 3 minutes each, applying maximum pestle pressure. A qualitative XRD scan was performed after grinding on a silicon sample holder. Mayenite was detected after the first burning and grinding step. Therefore, the powder was reheated to 1400 °C at a rate of 20 K/min with an 8-hour dwell time. Upon removal, the sample exhibited two different phase compositions: a grey/brown glass-like phase and a dense white powder phase. The material was divided into a white powder phase, a brown "glass" phase, and a mixed phase. The grinding procedure was repeated for all three sections, followed by qualitative XRD analysis, which showed identical phases for all samples. The reason for this uniformity is currently unknown. Additionally, no mayenite was observed after the second heating step. A quantitative XRD analysis was performed on the white powder phase with 20 wt.% ZnO. The samples were stored in parafilm-sealed snap-cap glass vials for two weeks in a desiccator until mixing.

After manufacturing the C_3_A, approximately 100 g of C_3_A was obtained. Friedel's salt was then synthesised by mixing C_3_A and CaCl_2_·2H_2_O in a 1:1 molar ratio. 48.6 g of C3A and 26.4 g of CaCl2·2H2O were measured in two bottles. For a water/solid ratio of 10, each bottle was filled with 750 ml of freshly distilled water and cooled to room temperature. The mixing was performed in an argon-filled glovebox. The bottles were mounted on an overhead mixer and rotated at 7 rpm for 30 days. The resulting solution was filtered using 598 Rundfilter (filter paper circles, d = 125 mm, Schleicher & Schuell), and the filtered solid was dried in a nitrogen-flushed desiccator over silica gel.

# Characterisation data:

## ^29^Si-NMR

| 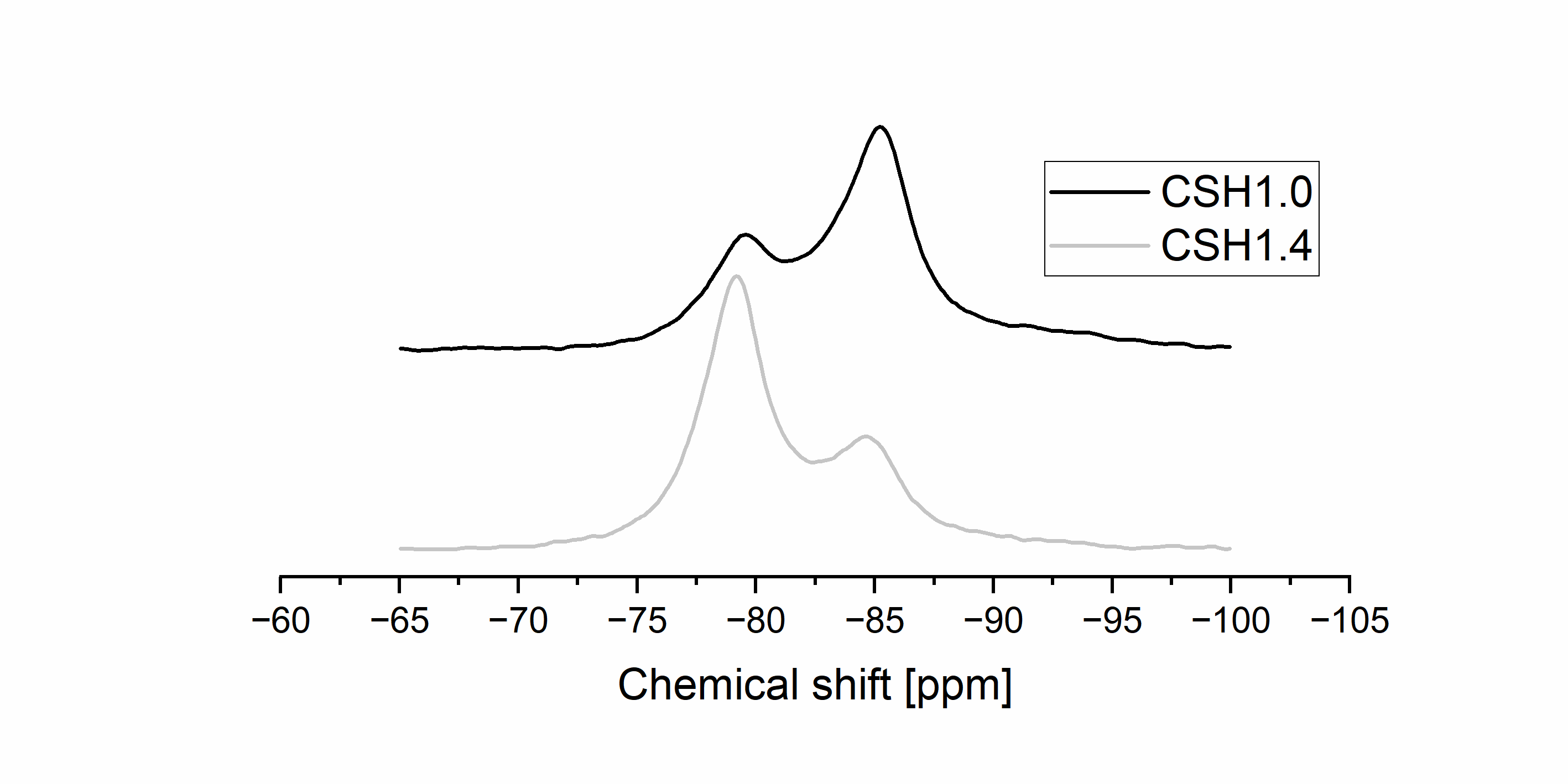 |
| --- |
| Figure S1 – ^29^Si-NMR plot of CSH1.0 (C/S = 1.0) and CSH1.4 (C/S = 1.0) |

| 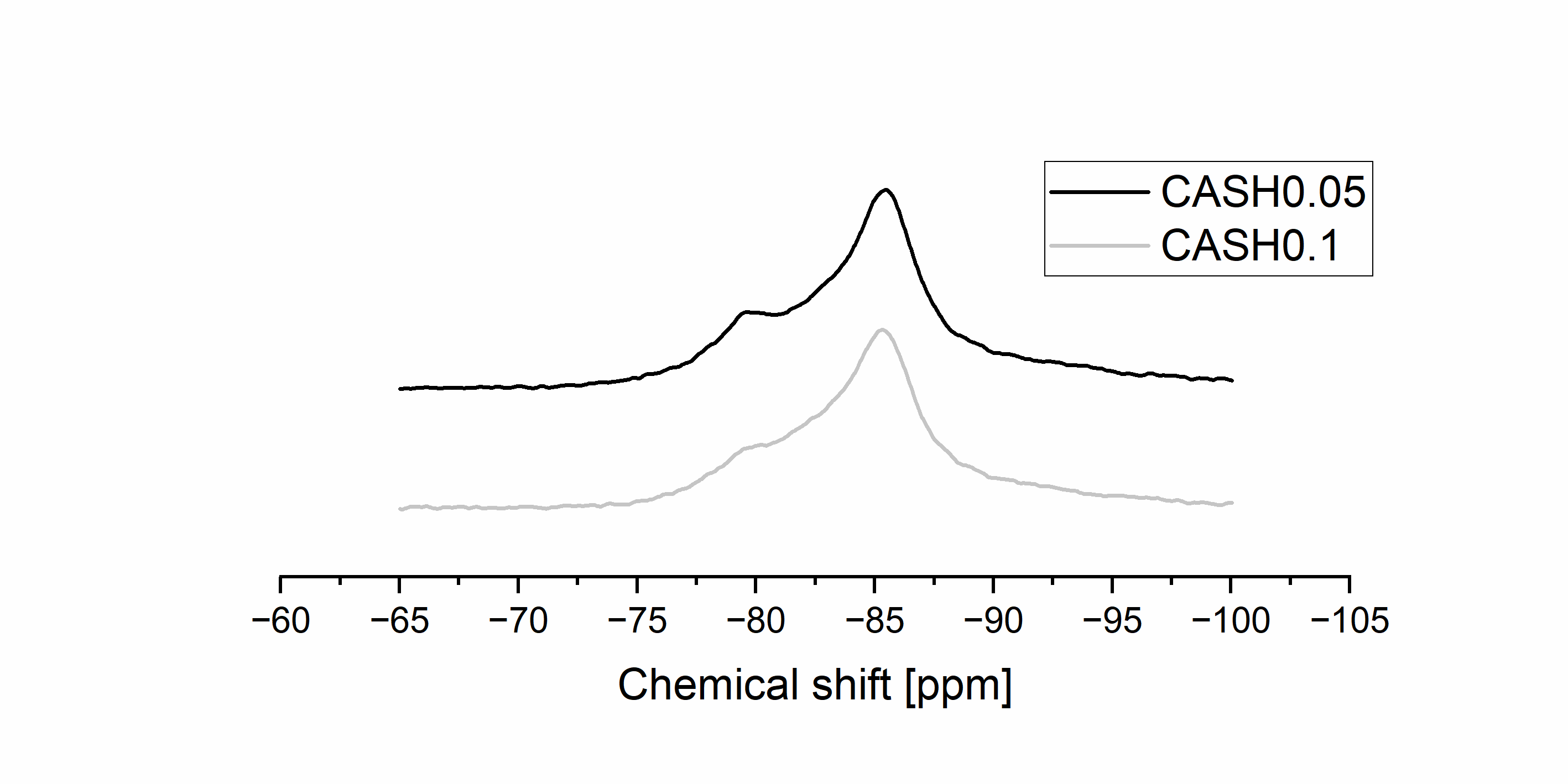 |
| --- |
| Figure S2 – ^29^Si-NMR plot of CASH0.05 (C/S = 1.0, A/S = 0.05) and CASH0.1 (C/S = 1.0, A/S = 0.1) |

## ^27^Al-NMR

| 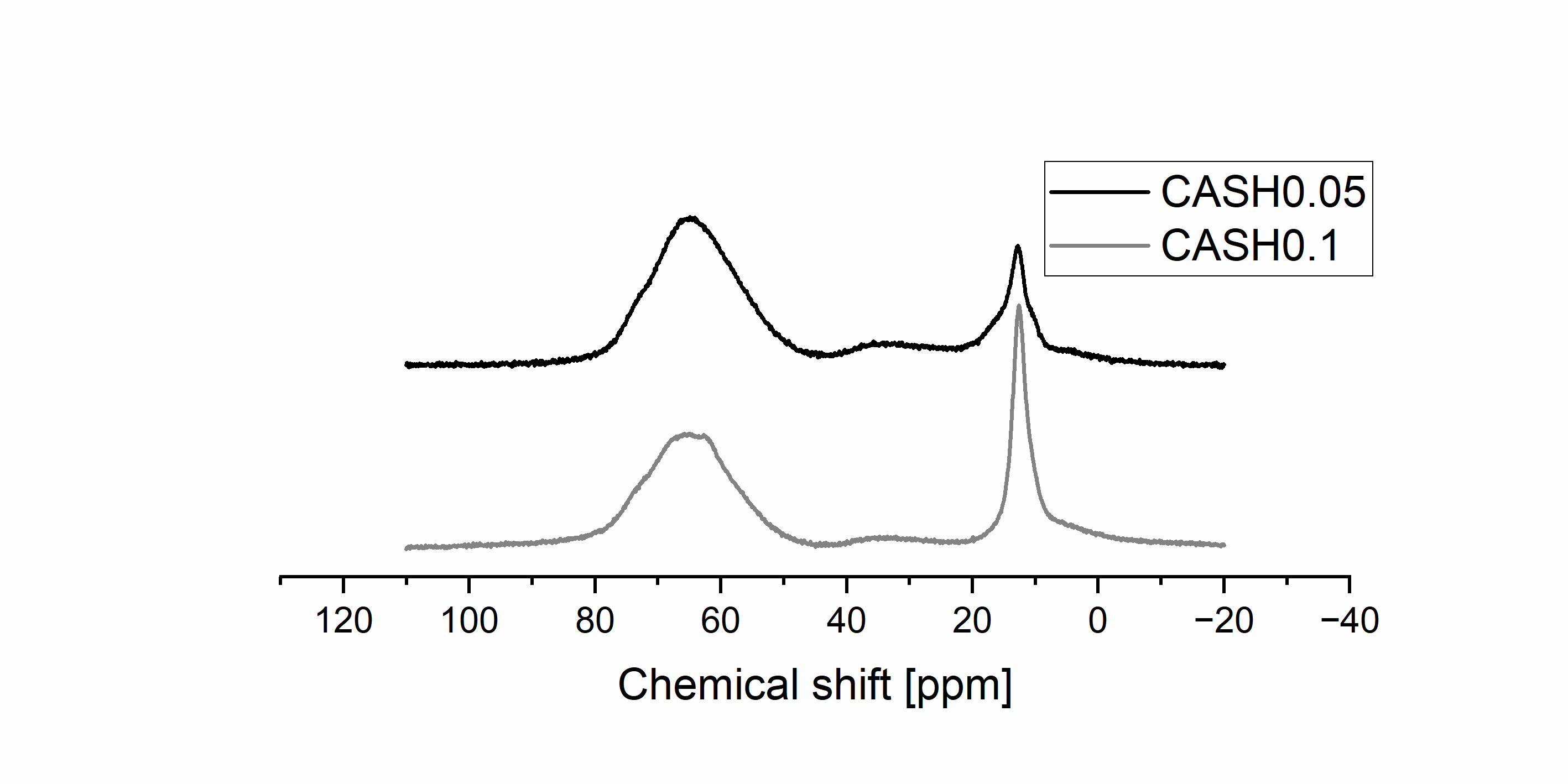 |
| --- |
| Figure S3 – ^27^Al-NMR plot of CASH0.05 (C/S = 1.0, A/S = 0.05) and CASH0.1 (C/S = 1.0, A/S = 0.1) |

| 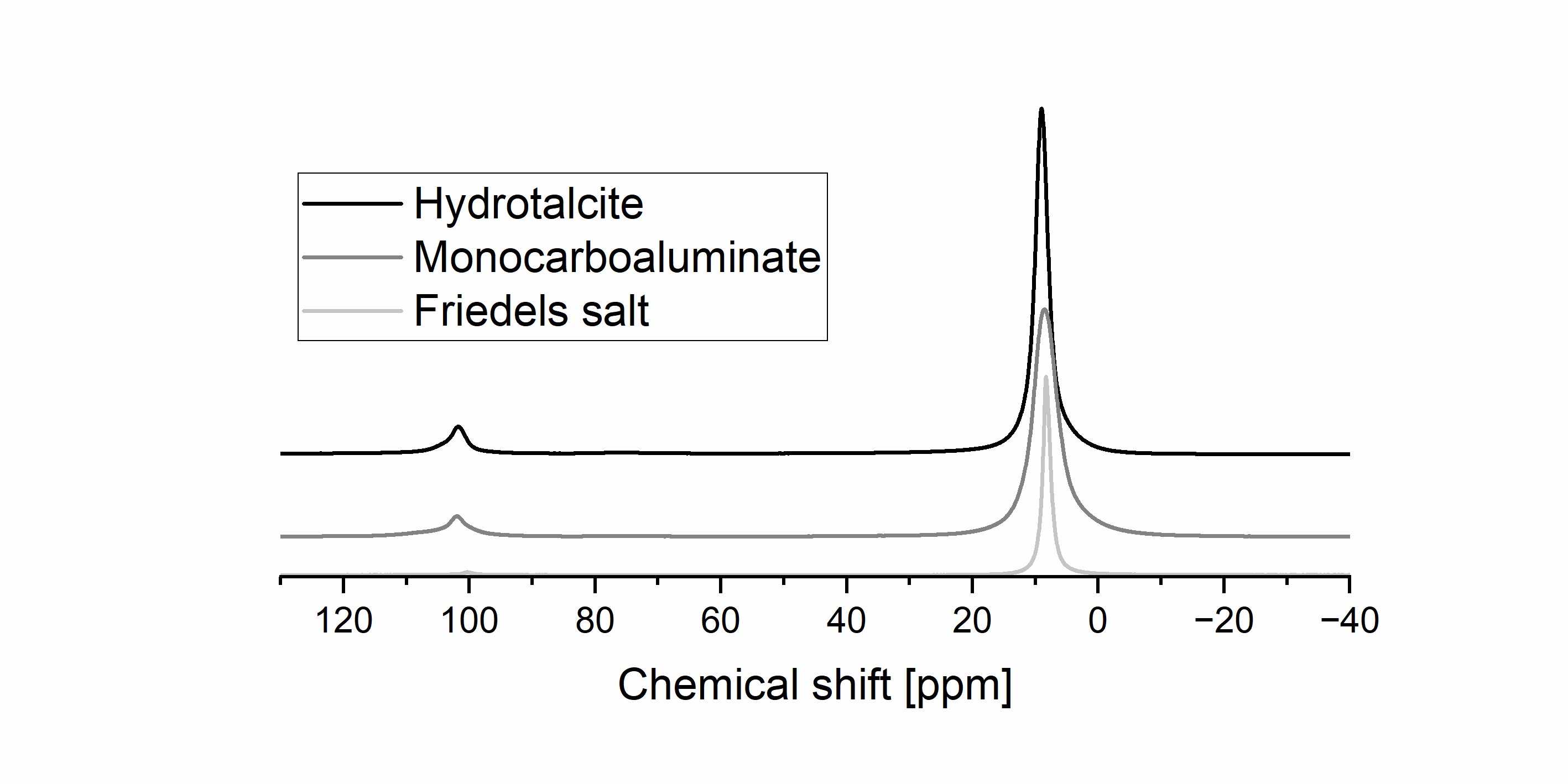 |
| --- |
| Figure S4 – ^27^Al-NMR plot of hydrotalcite, monocarboaluminate and Friedel’s salt |

## ICP-OES

Table S1 – ICP OES data for all synthesised phases

| **Sample** | **LoI** | **Na_2_O** | **K_2_O** | **CaO** | **MgO** | **Fe_2_O_3_** | **Al2O3** | **SiO2** | **SO3** | **Cl** |
| --- | --- | --- | --- | --- | --- | --- | --- | --- | --- | --- |
| **Dim** | **M%** | **M%** | **M%** | **M%** | **M%** | **M%** | **M%** | **M%** | **M%** | **M%** |
| CASH0.1 | 22.4 | 0.02 | 0.00 | 35.3 | 0.08 | 0.01 | 2.90 | 36.7 | 0.19 | - |
| CASH0.05 | 21.1 | 0.02 | 0.01 | 36.9 | 0.09 | 0.01 | 1.59 | 37.8 | 0.25 | - |
| Hydrotalcite | 44.2 | 0.32 | 0.03 | 0.1 | 34.4 | 0.01 | 19.7 | 0.82 | 0.05 | - |
| Monocarboaluminate | 41.1 | 0.24 | 0.50 | 35.9 | 0.15 | 0.03 | 21.3 | 1.07 | 0.05 | - |
| Friedel’s salt | 34.1 | 0.02 | 0.00 | 39.7 | 0.07 | 0.01 | 18.2 | 1.03 | 0.04 | 15.2 |
| CSH1.0 | 21.1 | 0.02 | 0.00 | 39.8 | 0.09 | 0.01 | 0.06 | 38.0 | 0.06 | - |
| CSH1.4 | 24.3 | 0.02 | 0.00 | 44.1 | 0.11 | 0.01 | 0.06 | 31.5 | 0.10 | - |

## XRD

| 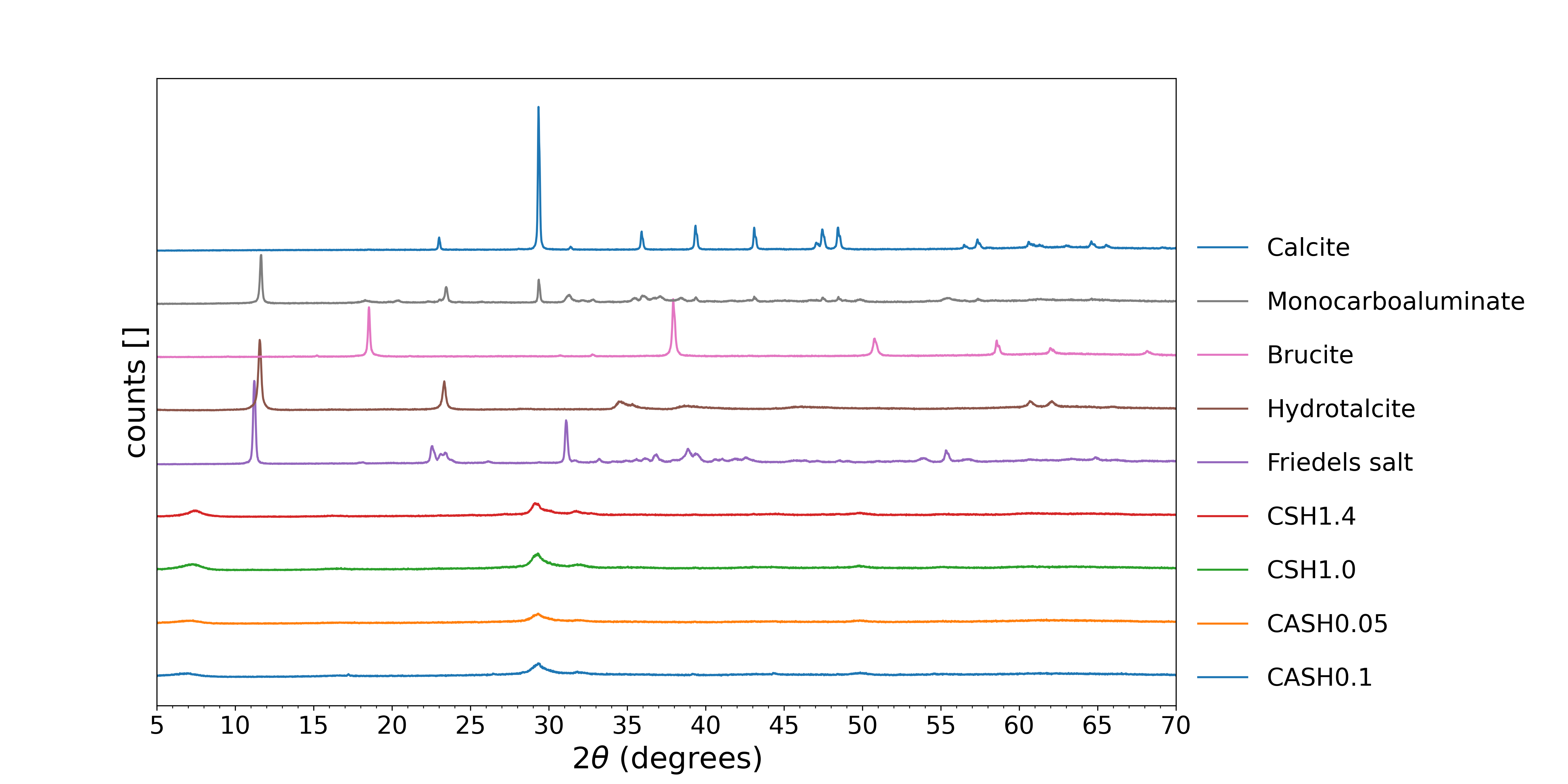 |
| --- |
| Figure S5 – Qualitative XRD from all synthesised phases |
| 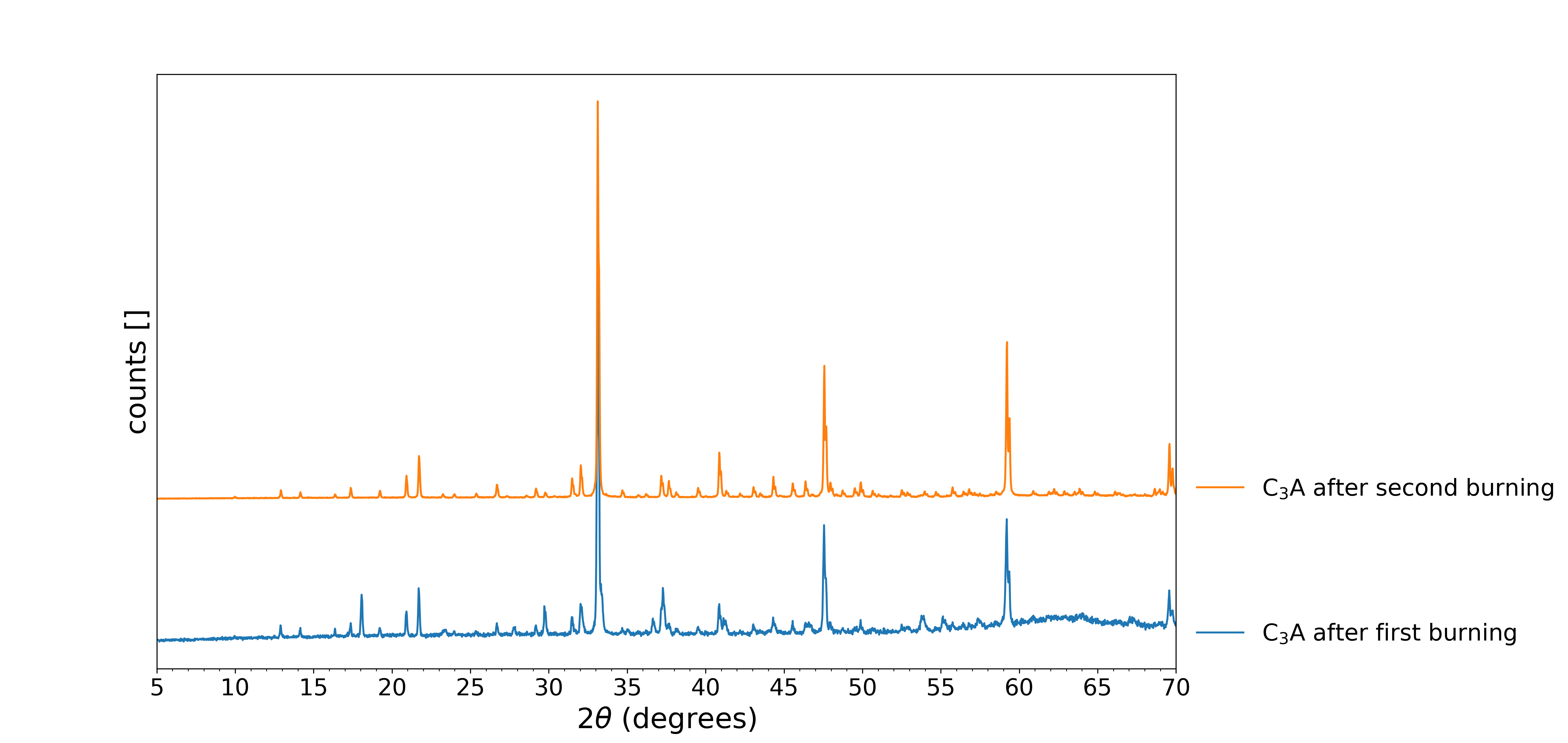 |
| Figure S6 – Qualitative XRD from C_3_A synthesis |

## FTIR

| 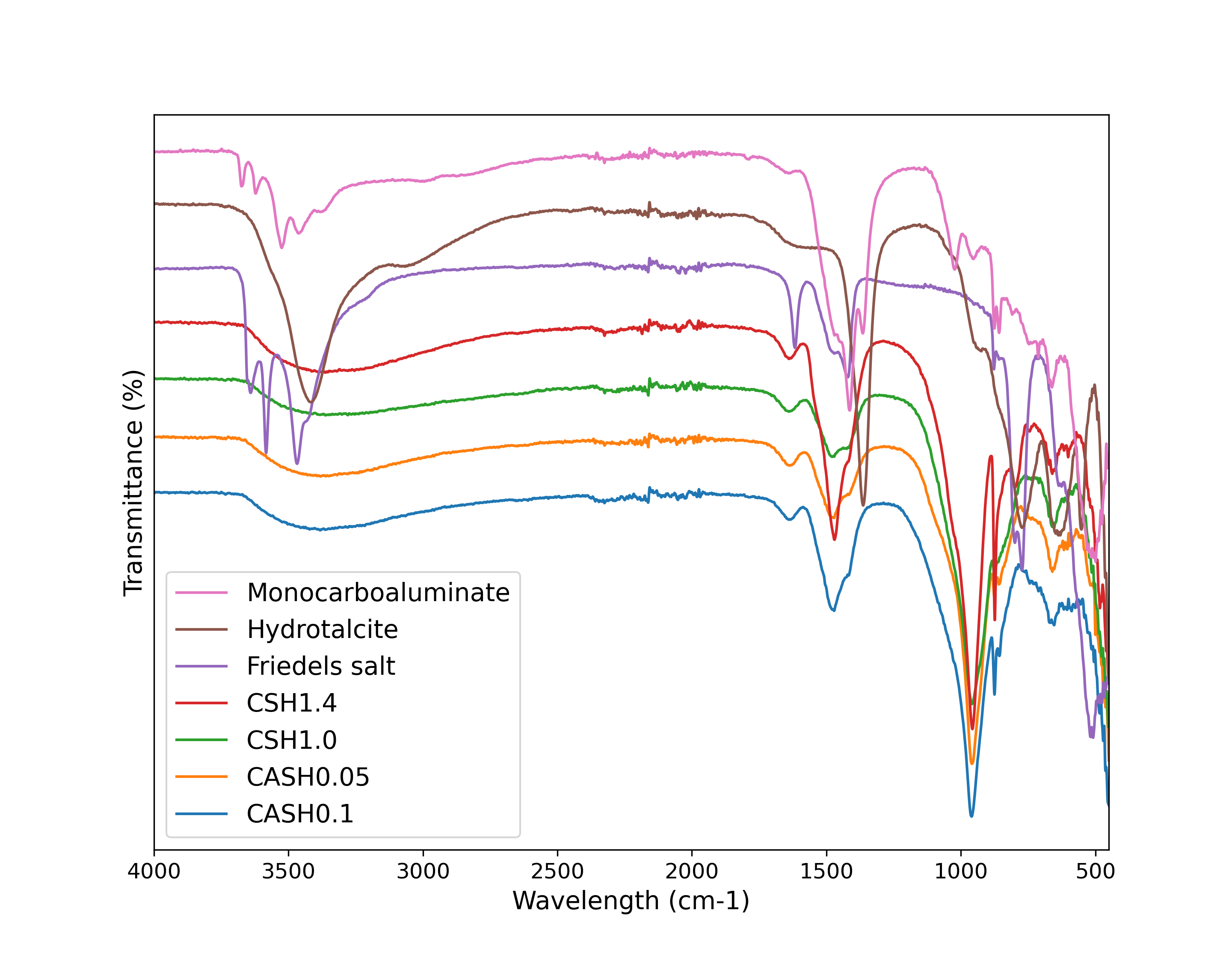 |
| --- |
| Figure S7 – FTIR of all synthesized phases |

## TGA

| 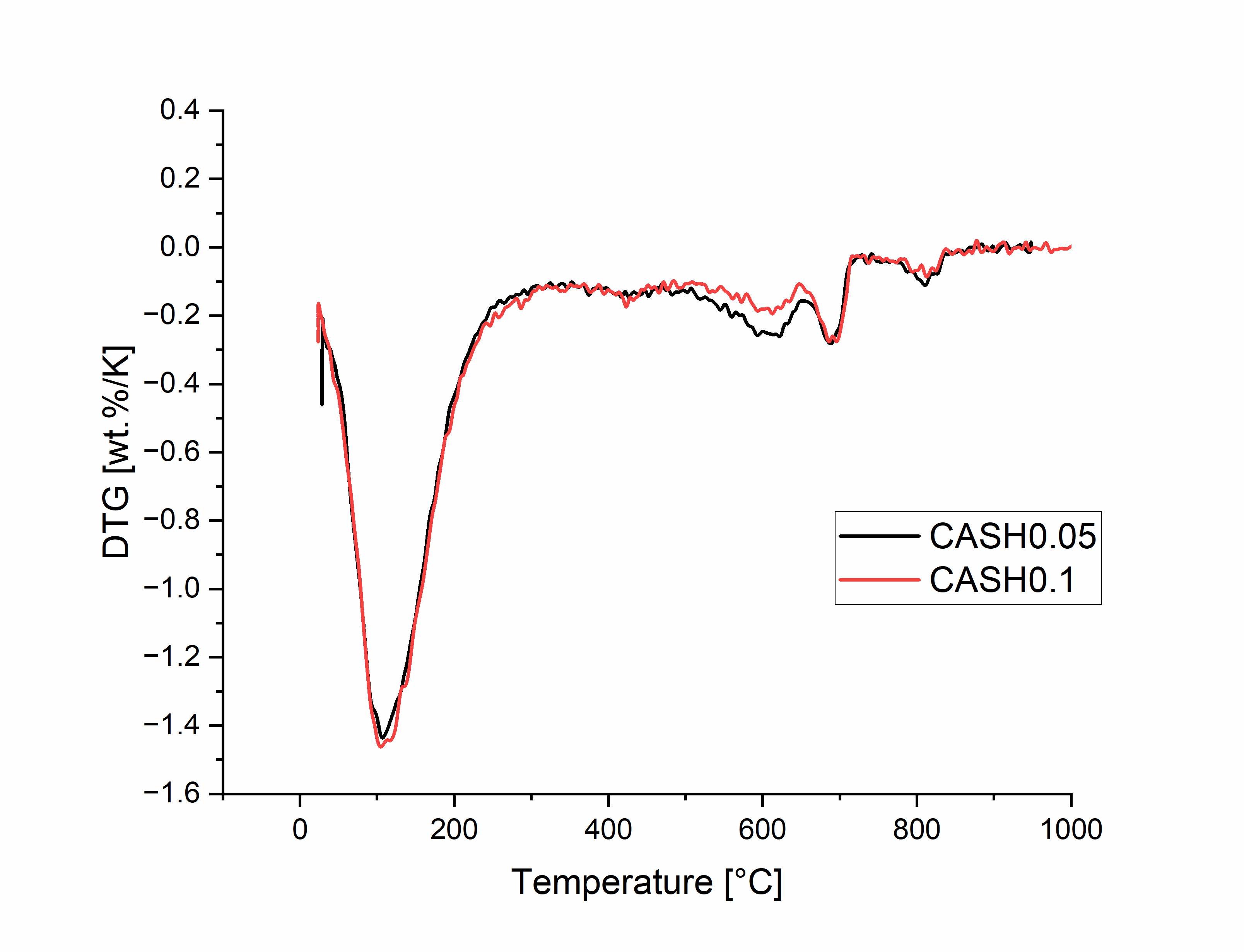 | |
| --- | --- |
| Figure S8 - Thermogravimetric analysis (DTG) for CASH0.05 and CASH0.1 | |
| 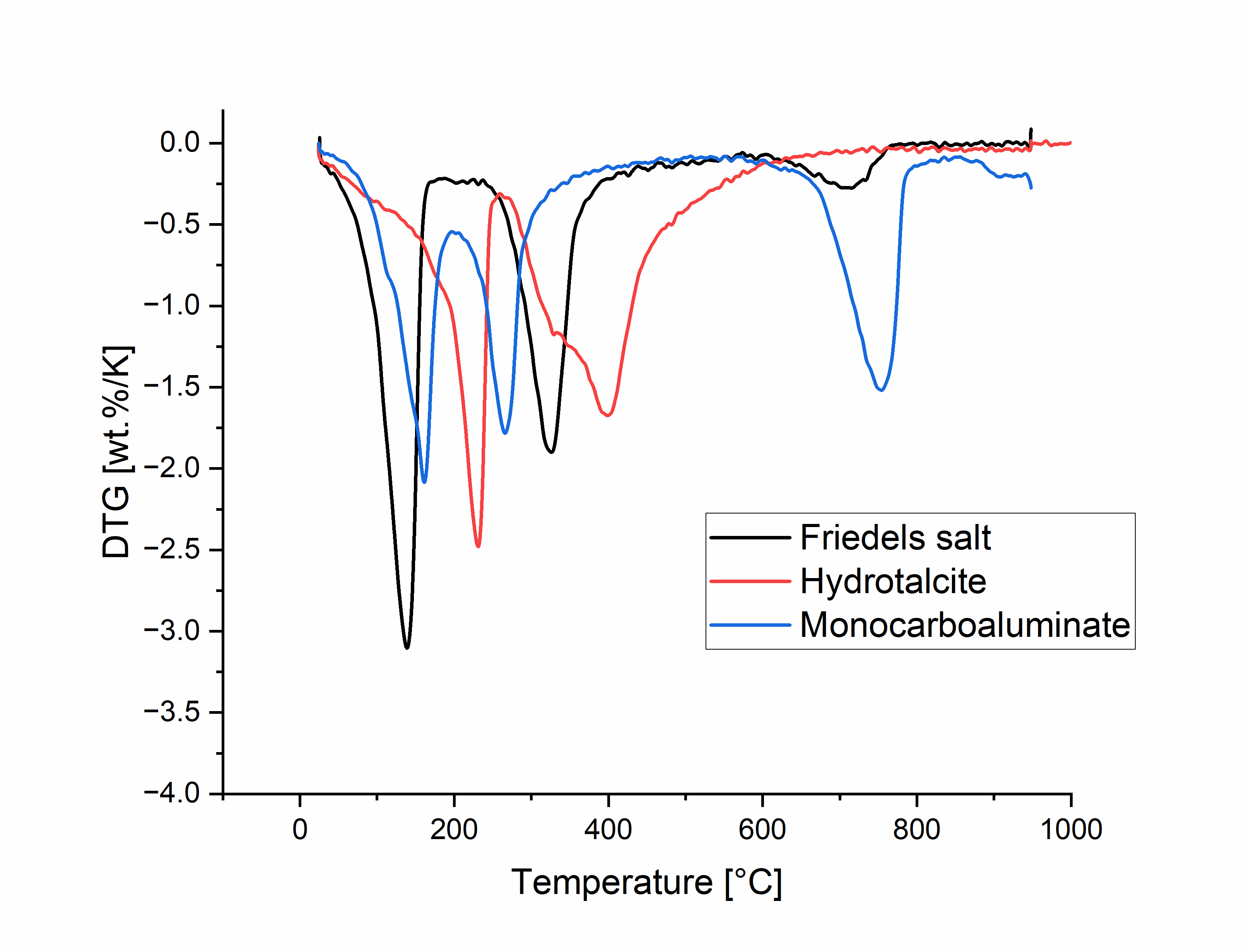 |  |
| Figure S9 – Thermogravimetric Analysis for hydrotalcite, monocarboaluminate, and Friedel’s salt |  |

# Additional decolourisation data

| 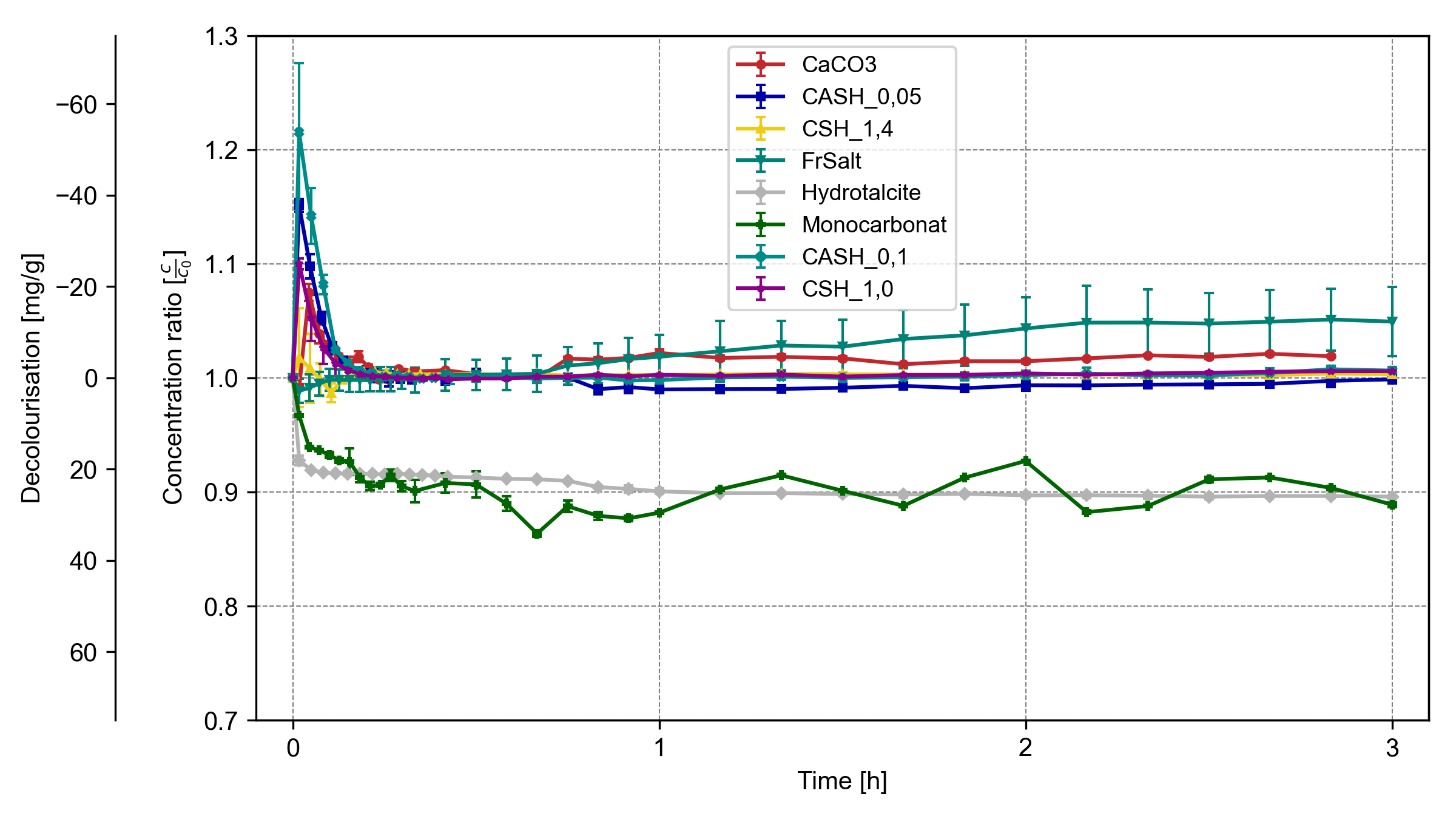 |
| --- |
| Figure S10 – Decolourization data for the dye Acid Green 1. Brucite is not shown as the aquired data was not reproducable |

| 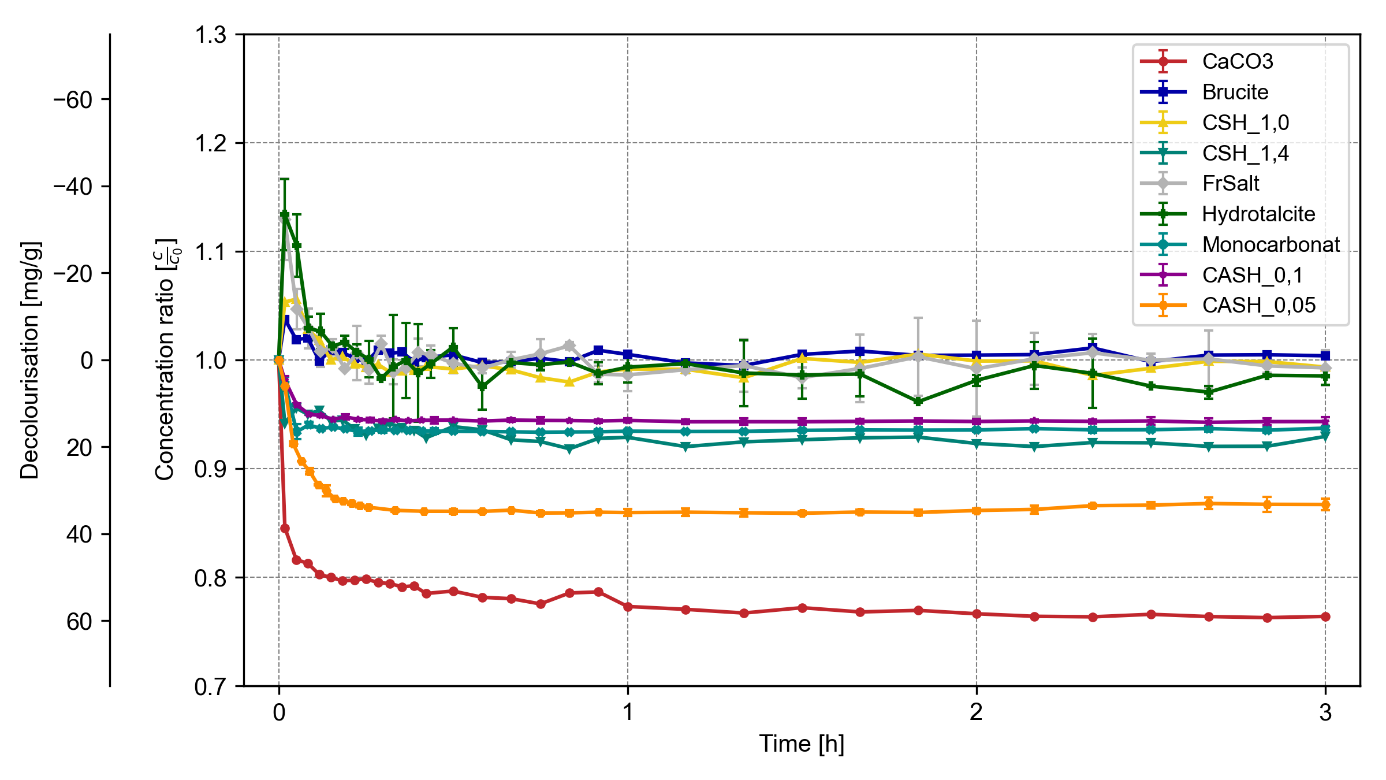 |
| --- |
| Figure S11 – Decolourization data for the dye Acid Orange 7. |

# Post-decolourisation data:

| 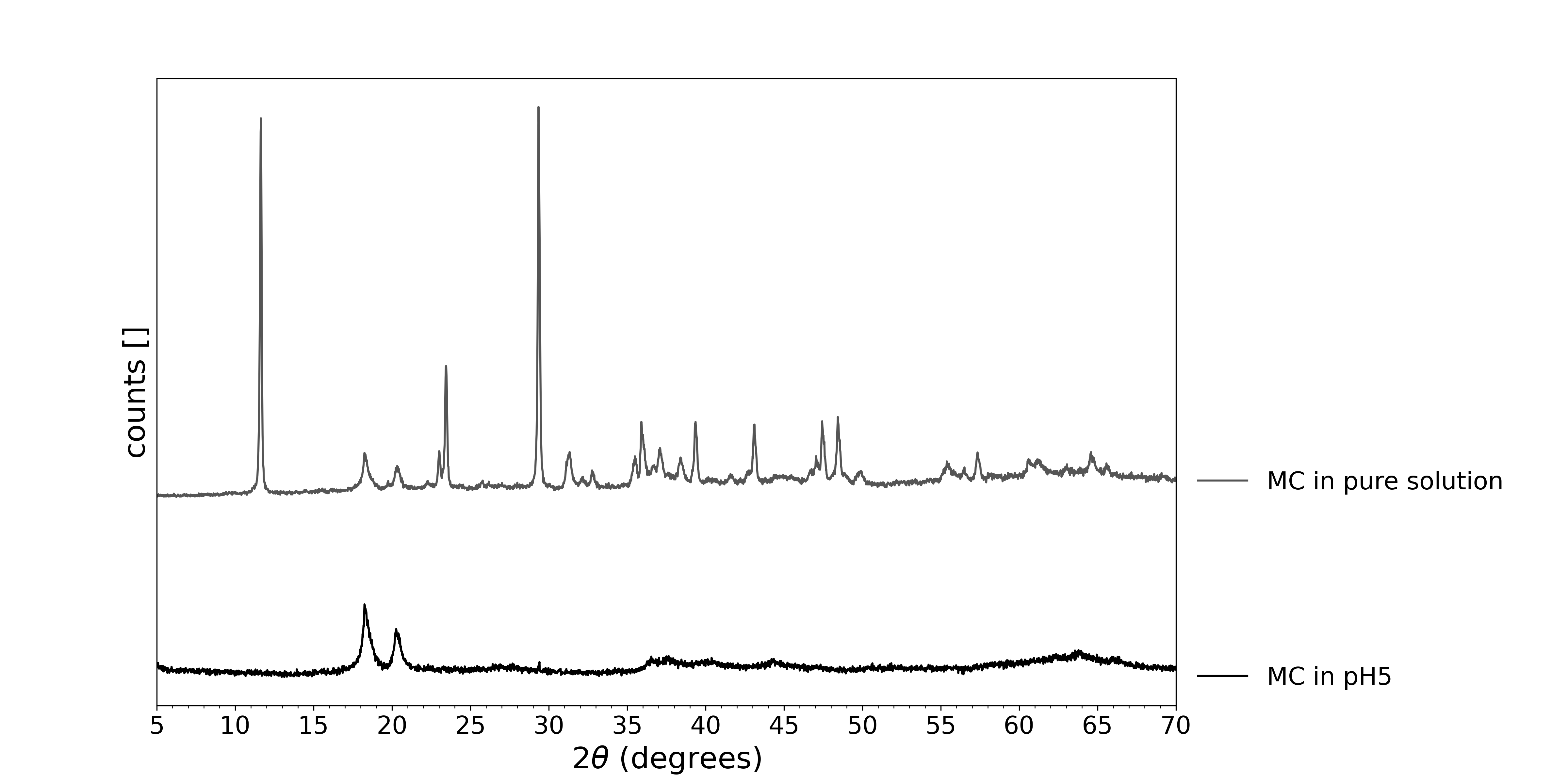 |
| --- |
| Figure S12 – XRD of Monocarboaluminate after pH 5 decolourisation |

| 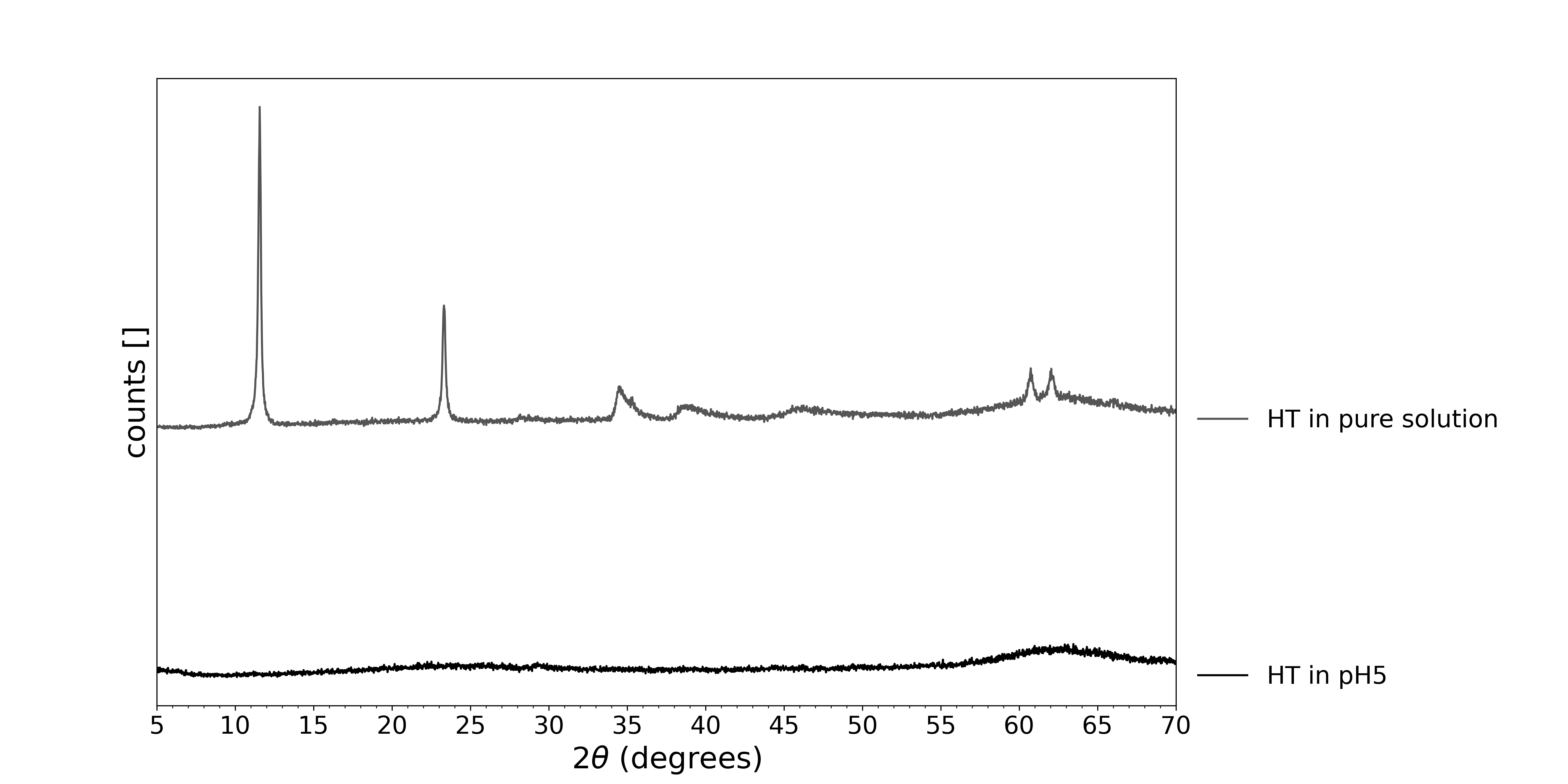 |
| --- |
| Figure S13 – XRD of Hydrotalcite after pH 5 decolourisation |

| 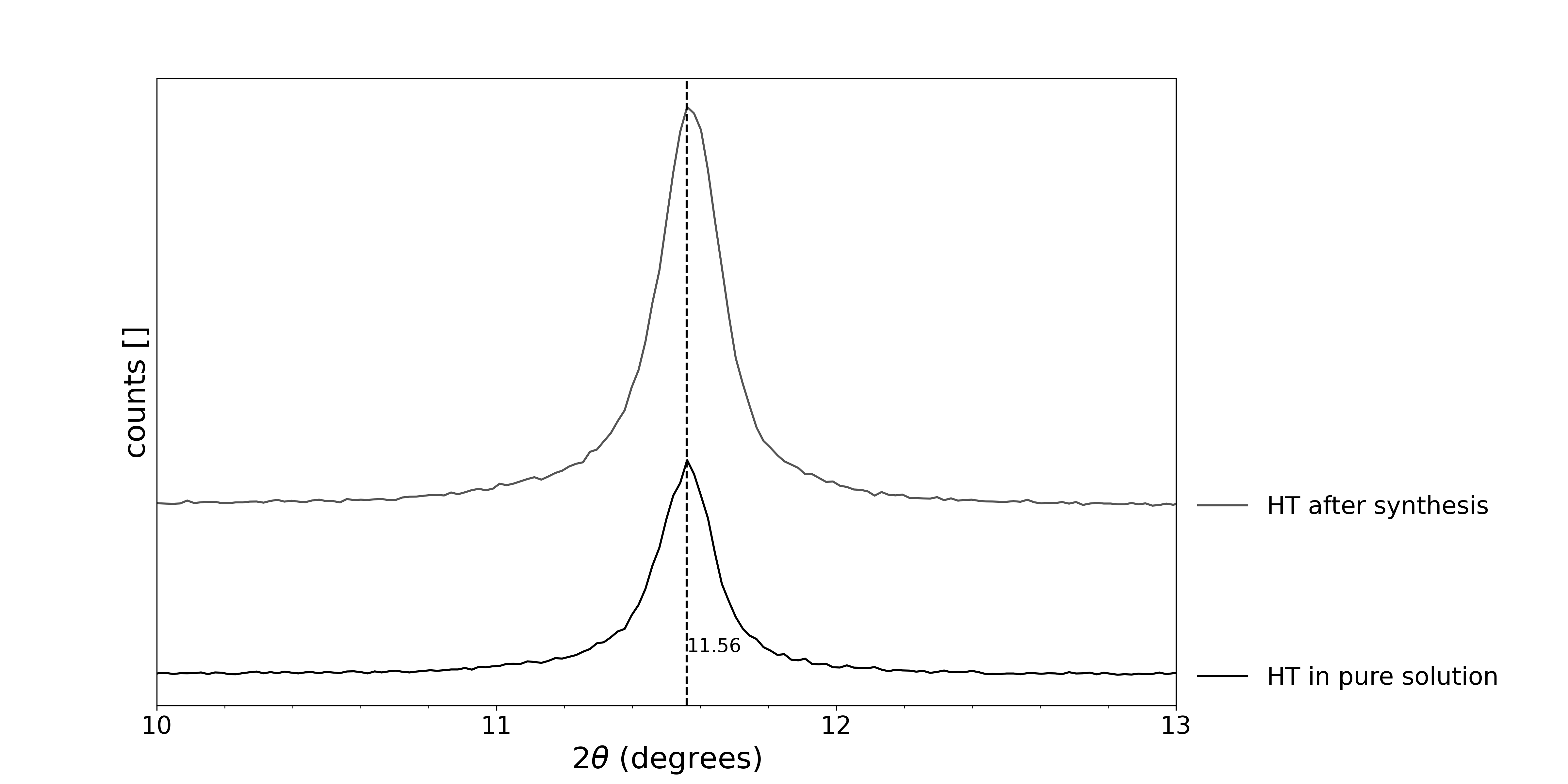 |
| --- |
| Figure S14 - XRD of hydrotalcite before and after the decolourisation experiment from 10 - 13 2°Theta |
